# Enhancing the Catalytic Activity of Pd Nanocatalysts for Anion Exchange Membrane Direct Ethanol Fuel Cells by Functionalizing Vulcan XC-72 with Cu Organometallic Compounds

**DOI:** 10.1021/acsanm.4c02670

**Published:** 2024-08-20

**Authors:** P. C. Meléndez-González, M. O. Fuentez-Torres, M. E. Sánchez-Castro, I. L. Alonso-Lemus, B. Escobar-Morales, W. J. Pech-Rodríguez, Teko W. Napporn, F. J. Rodríguez-Varela

**Affiliations:** † Nanociencias y Nanotecnología, Cinvestav Unidad Saltillo, Av. Industria Metalúrgica 1062, Parque Industrial Ramos Arizpe, Ramos Arizpe, Coahuila C.P 25900, México; ‡ Sustentabilidad de Los Recursos Naturales y Energía, Cinvestav Unidad Saltillo, Ramos Arizpe, Coahuila C.P 25900, México; § CONAHCYT-Cinvestav Saltillo, Sustentabilidad de Los Recursos Naturales y Energía, Cinvestav Unidad Saltillo. Av. Industria Metalúrgica 1062, Parque Industrial Ramos Arizpe. Ramos Arizpe, Coahuila C.P 25900, México; ∥ CONAHCyT, Centro de Investigación Científica de Yucatán, Unidad de Energía Renovable, Calle 43, No. 130 Col. Chuburná de Hidalgo, Mérida, Yucatán C.P. 97200, México; ⊥ Universidad Politécnica de Victoria, Parque Científico y Tecnológico de Tamaulipas, Av. Nuevas Tecnologías 5902, Cd Victoria, Tamaulipas C.P. 87138, México; # Université de Poitiers, IC2MP UMR 7285 CNRS, ⟨⟨Equipe SAMCat⟩⟩, 4, Rue Michel Brunet, B27, TSA 51106, Poitiers Cedex 09 86073, France

**Keywords:** surface functionalization, Cu organometallic compounds, Pd nanocatalysts, ethanol oxidation reaction, anion exchange membrane direct ethanol fuel cells

## Abstract

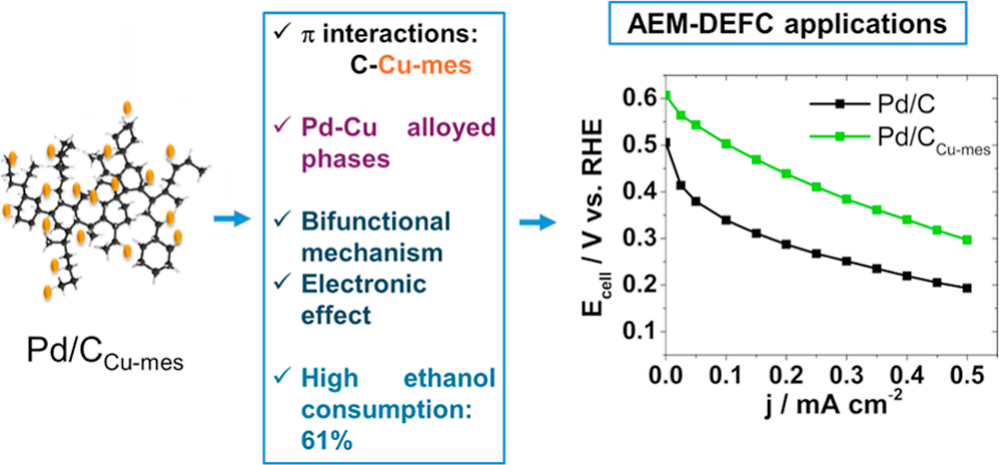

The most widely used support in low-temperature fuel cell applications is the commercially available Vulcan XC-72. Herein, we report its functionalization with the home-obtained mesityl copper (Cu-mes) and Cu coordinate (Cu(dmpz)L2) organometallic compounds. Pd nanoparticles are anchored on the supports obtaining Pd/C_Cu-mes_, Pd/C_Cu(dmpz)L2_, and Pd/C (on nonfunctionalized support). The polarization curves of the ethanol oxidation reaction (EOR) show that Pd/C_Cu-mes_ and Pd/C_Cu(dmpz)L2_ promote the reaction at a more negative onset potential, i.e., *E*_onset_ = 0.38 V/reversible hydrogen electrode (RHE), compared to 0.41 V/RHE of Pd/C. The mass current density (*j*_m_) delivered by Pd/C_Cu-mes_ is considerably higher (1231.3 mA mg_Pd_^–1^), followed by Pd/C_Cu(dmpz)L2_ (1001.8 mA mg_Pd_^–1^), and Pd/C (808.3 mA mg_Pd_^–1^). The enhanced performance of Pd/C_Cu-mes_ and Pd/C_Cu(dmpz)L2_ for the EOR (and tolerance to CO poisoning) is attributed to a shift of their d-band center toward more negative values, compared to Pd/C, because of the formation of PdCu alloyed phases arising from the functionalization. In addition, laboratory-scale tests of the anion exchange membrane-direct ethanol fuel cell assembled with Pd/C_Cu-mes_ show the highest open circuit voltage (OCV = 0.60 V) and cell power density (*P*_cell_ = 0.14 mW cm^–2^). As a result of its high catalytic activity, Pd/C_Cu-mes_ can find application as an anode nanocatalyst in AEM-DEFCs.

## 1 Introduction

Over the past decade, anion exchange membrane direct ethanol fuel cells (AEM-DEFCs) have emerged as promising devices for clean energy generation.^1^ AEM-DEFCs have attracted great attention from international scientific groups because of their fast electrochemical reactions and lower corrosion risk compared to the same type of cells operating with acid membranes.^2^ Moreover, there is the attractiveness of using ethanol as fuel, instead of other liquid alcohols, since this molecule has a high theoretical energy density and offers the advantage of being obtained from biomass as an alternative to conventional processes.^3^

However, there exists the technological need to increase the overall performance and thus the efficiency of AEM-DEFCs.^4−6^ One of the most practical approaches to achieve this is developing stable nanocatalysts with enhanced catalytic activity for the reactions occurring therein. Typically, carbon-supported semispherical nanoparticles have a high electrochemically active surface area (ECSA) available for the ethanol oxidation reaction (EOR), thus showing enhanced catalytic properties for more efficient fuel cell performances.^7,8^ Additionally, as indicated by the US Department of Energy (DOE), having electrochemically stable fuel cell nanocatalysts is of utmost importance for advancing their commercialization.^9^

Vulcan XC-72 is the most commonly used carbonaceous support for fuel cell nanoparticles, since it has suitable surface chemistry for anchoring them with a proper dispersion, along with a high electrical conductivity.^10^ Nevertheless, being relatively hydrophobic, Vulcan XC-72 has been submitted to diverse oxidative functionalizing treatments, which in several cases end-up decreasing its electrical conductivity.^11,12^

On this matter, a family of organometallic compounds have emerged as less aggressive functionalizing agents than their oxidative counterparts, but strong enough to chemically modify the carbon surface developing functional groups which facilitate the anchorage and homogeneous dispersion of metal nanoparticles.^13,14^ Furthermore, this novel approach for surface modification creates organometallic active metal sites that promote the development of alloyed phases with the main metal (e.g., Pt and Pd) deposited on the functionalized carbon.

From X-ray photoelectron spectroscopy (XPS) analysis, shifts in binding energy (BE) of the Pt^0^ species in the Pt 4f_7/2_ state in the order of 0.32–0.96 eV have been determined when functionalizing Vulcan with Ru organometallic compounds, compared to nonfunctionalized carbon support, confirming the development of Pt–Ru alloyed phases. Moreover, Ru and RuO_2_ species have been identified, providing the supported Pt–Ru alloyed nanocatalysts with higher catalytic activity for the oxidation of methanol than monometallic Pt/C due to the bifunctional mechanism.^13,14^ Even more, when functionalizing reduced graphene oxide with Cr compounds, a shift of ca. 0.2 eV of the Pt^0^ species has been obtained (also compared to nonmodified support), along with the promotion of CrO_2_, Cr_2_O_3_, and CrO_3_ species, improving the catalytic activity of nanocatalysts supported on functionalized carbon for the oxygen reduction reaction (ORR).^15^

In a similar fashion, Pd alloys show high catalytic activity for EOR in alkaline media. The Pd_2_Ru/C nanocatalyst in^7^ outperforms Pd/C for the EOR, favoring the formation of acetaldehyde and the oxidation of CO. The good performance of Pd_2_Ru/C in an AEM-DEFC has been attributed to its high electronic vacancies and the bifunctional mechanism, nevertheless, dropping in performance during stability tests due to strong acetaldehyde adsorption. The Pd_41_Au_29_Ni_30_/C nanocatalyst in^16^ surpasses the catalytic activity, stability, and performance in AEM-DEFC of Pd/C due to improved CO tolerance and a synergistic effect of Pd, Au, and Ni.

In this context, the modification of Pd with cheap transition metals, such as Cu, also increases its electrochemical performance due to a synergistic effect between the two metals. For instance, a functional method to obtain web-footed PdCu nanosheets with high catalytic activity for both ethanol and formic acid oxidation reactions has been reported recently.^17^ The performance of the PdCu catalysts has been attributed to the tuned electronic structure and geometric configuration of the nanosheets. Moreover, a facile strategy to selectively etching Pd to create edge sites, improving the surface area and reducing the charge transfer resistance has been introduced, resulting in an enhanced catalytic activity of the catalysts for the oxidation of formic acid.^18^ Therefore, the Pd–Cu alloying is of special interest in this work because of its higher performance for the EOR related to monometallic Pd, ascribed to improved tolerance to CO, enhanced stability, electronic modulation of Pd structure, and the bifunctional mechanism.^19−22^ Moreover, organometallic Cu compounds have been classified as high valence complexes because of their ability to form Cu–C bonds, due to their variable oxidation states (1+, 2+, and 3+). Moreover, such compounds have the advantage of their relatively easy synthesis, in addition to their easiness to form carbon–carbon and carbon-heteroatoms bonds.^23^ On this context, Cu-mes and Cu(dmpz)L2 have the advantage of being compounds with good solubility and adequate chemical stability.

In this work, the synthesis of Cu-mes and Cu(dmpz)L2 organometallic compounds is reported in detail. From there, a novel approach using these compounds as functionalizing agents of Vulcan (identified as C) is introduced, giving rise to the C_Cu-mes_ and C_Cu(dmpz)L2_ supports. The aim of such functionalization is to develop functional groups and Cu metal sites on the surface of C. Functional groups and the Cu atoms are intended to act as sites for the anchorage of Pd nanoparticles. Indeed, the development of Cu atoms is purposely done to form PdCu alloyed phases when the supports are used to synthesize the nanocatalysts labeled as Pd/C_Cu-mes_ and Pd/C_Cu(dmpz)L2_, with the objective of enhancing their catalytic activity.

This approach differs from our previous report on the implementation of Cu organometallic compounds^24^ in the type of noble metal (Pt there, Pd here) and electrochemical reaction (ORR and OER in that report, EOR in this work). Therefore, to the best of our knowledge, this is the first time that such Cu compounds are used to functionalize C and develop Pd-based nanocatalysts for the EOR. The nanocatalysts are evaluated as anodes for the EOR in alkaline media. Furthermore, their performance in an AEM-DEFC is evaluated.

## 2 Experimental Section

The chemical reagents were of analytical grade: Ammonium hexachloropalladate (IV) (PdCl_6_(NH_4_)_2_) (99%), ethylene glycol (EG) (C_2_H_6_O_2_) (99.8%), sulfuric acid (H_2_SO_4_) (95–99%), sodium hydroxide (NaOH) (97%), copper(I) chloride (CuCl (I)) (99.9%), 1,4-dioxano (C_4_H_8_O_2_) (99.8%), ethylic ether ((C_2_H_5_)_2_O) (99%), 3,5 dimethyl pyrazole (C_5_H_8_N_2_) (99%), copper(II) acetate monohydrate (Cu(CO_2_CH_3_)_2_·H_2_O) (99%), terephthalic acid (C_8_H_6_O_4_) (97.5%), tetrahydrofuran (THF, C_4_H_8_O) (99.9%), potassium hydroxide (KOH) (90%), deuterated acetone ((CD_3_)_2_CO) (99.98%), benzene (C_6_D_6_) (99.99%), Nafion solution (5 wt %), 2-propanol (C_3_H_8_O) (95%), and ethanol (C_2_H_5_OH) (99.8%) were purchased from Sigma-Aldrich. Metallic sodium (Na^0^) and deionized water were acquired from Jalmeck. Vulcan XC-72 was obtained from Cabot. UHP Ar and O_2_ were purchased from Infra (purity >99%).

### 2.1 Synthesis of Mesitylcopper (Cu-mes)

The preparation of the Cu-mes was carried out under an Ar atmosphere using a Schlenk line. The glassware was previously washed and heated at 95 °C overnight to eliminate moisture. The solvents (dioxane, THF, and ether) were distilled before use with Na^0^ in refluxing conditions by 2 h, to avoid the formation of metallic oxides.

A suspension of CuCl (I) (10.9 g, 110 mmol) in THF (100 mL) was ultrasonically mixed for 1 h. Then 100 mL of mesityl BrMg were added under vigorous magnetic stirring for 12 h at room temperature in Ar atmosphere. A change in color from green to brown was observed. Dioxane (50 mL) was added to the mixture, maintaining stirring for 2 h, allowing precipitation for 1 h. The BrMgCl salt obtained had a white color.

The recovered yellow liquid was mixed with 50 mL of ether, and the solution was allowed to precipitate for 30 min. The resulting crystals were filtered and dried in vacuum to obtain Cu-mes as yellow/brown powder with 43.79% (6 g, 32 mmol) reaction yield.

### 2.2 Synthesis of the Cu Compound Coordinated with 3,5 Dimethylpyrazole (dmpz) and Terephthalic Acid (L2) (Cu(dmpz)L2)

**Scheme 1:**
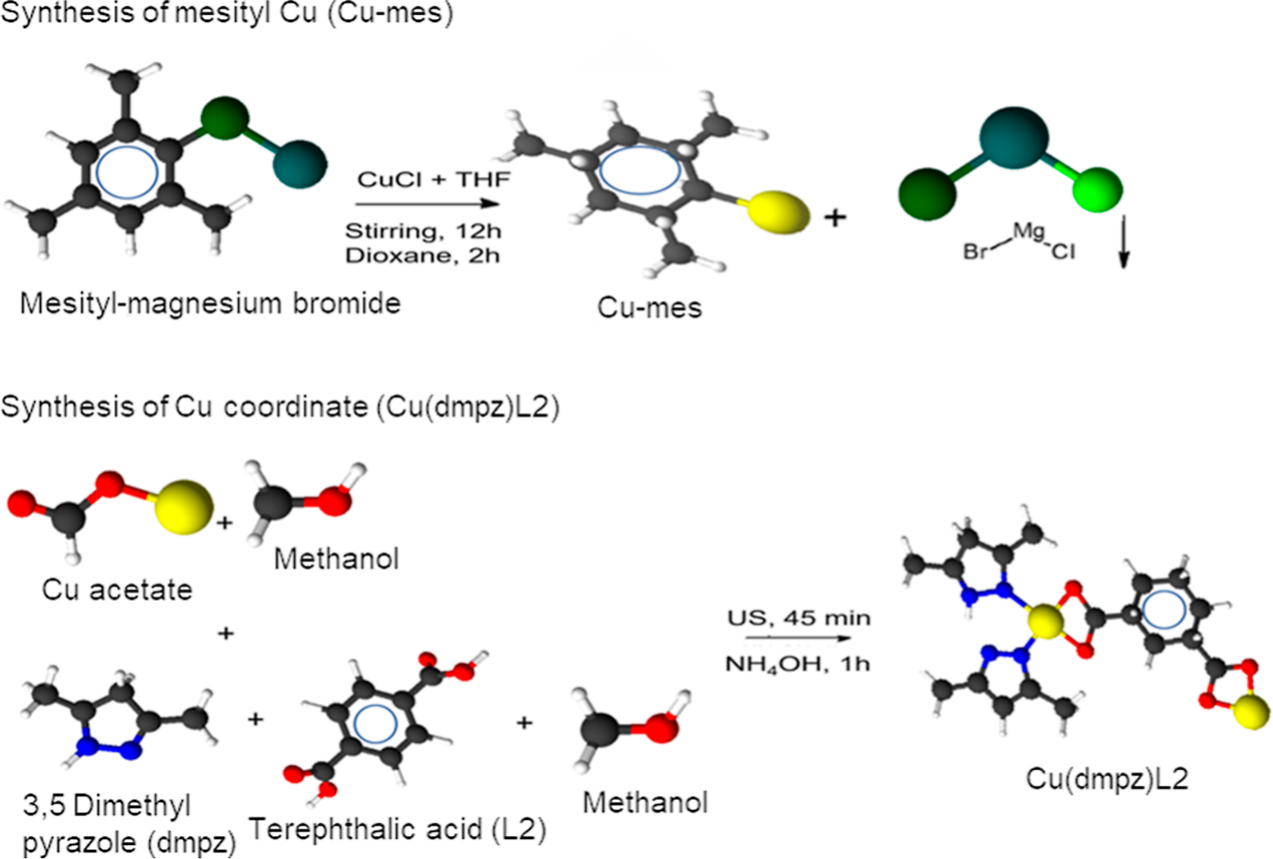
Synthesis of the Cu-mes and Cu(dmpz)L2 Compounds

Cu(dmpz)L2 was synthesized as follows: a solution of copper(II) acetate (0.199 g) in methanol (15 mL) was mixed with 12 mL of methanol containing dmpz (0.192 g) and terephthalic acid (L2) (0.28 g) under continuous stirring. The resulting solution was dispersed for 45 min by ultrasound at room temperature. Afterward, 1 mL of NH_4_OH was added, allowing precipitation for 1 h. The color changed from blue/green to purple. The resulting product was filtered, washed with methanol, and dried to obtain Cu(dmpz)L2 as a blue powder with 77.7% (0.7 g) reaction yield. Scheme 1 shows the synthesis of both Cu-mes and Cu(dmpz)L2.

### 2.3 Functionalization of Vulcan (C)

**Scheme 2:**
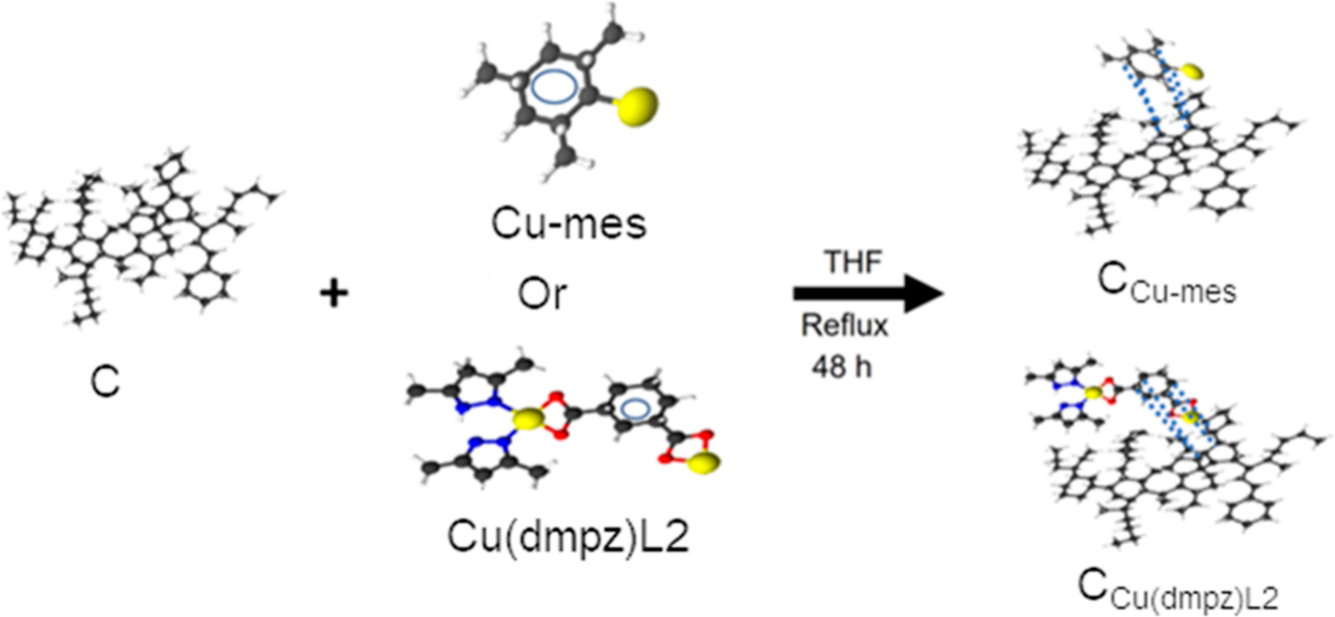
Functionalization of C to Produce the C_Cu-mes_ and C_Cu(dmpz)L2_ Supports

C_Cu-mes_ was obtained from the functionalization of Vulcan XC-72. 0.4 g of Cu-mes and 0.6 g of C (Cu-mes: C molar ratio of 1:10) were stirred in 80 mL of THF under an Ar atmosphere and refluxing conditions for 48 h at 130 °C. The black solution generated was transferred into a Schlenk tube, filtered through a cannula, and washed with dried THF, ethanol, and water. The product was finally dried in a vacuum for 3 h, resulting in a black powder. C_Cu(dmpz)L2_ was obtained following the same procedure using 0.7 g of Cu(dmpz)L2 and 0.2 g of C, i.e., the same molar ratio. Scheme 2 shows the procedure for the functionalization of C.

### 2.4 Synthesis of the Pd Nanocatalysts

**Scheme 3:**
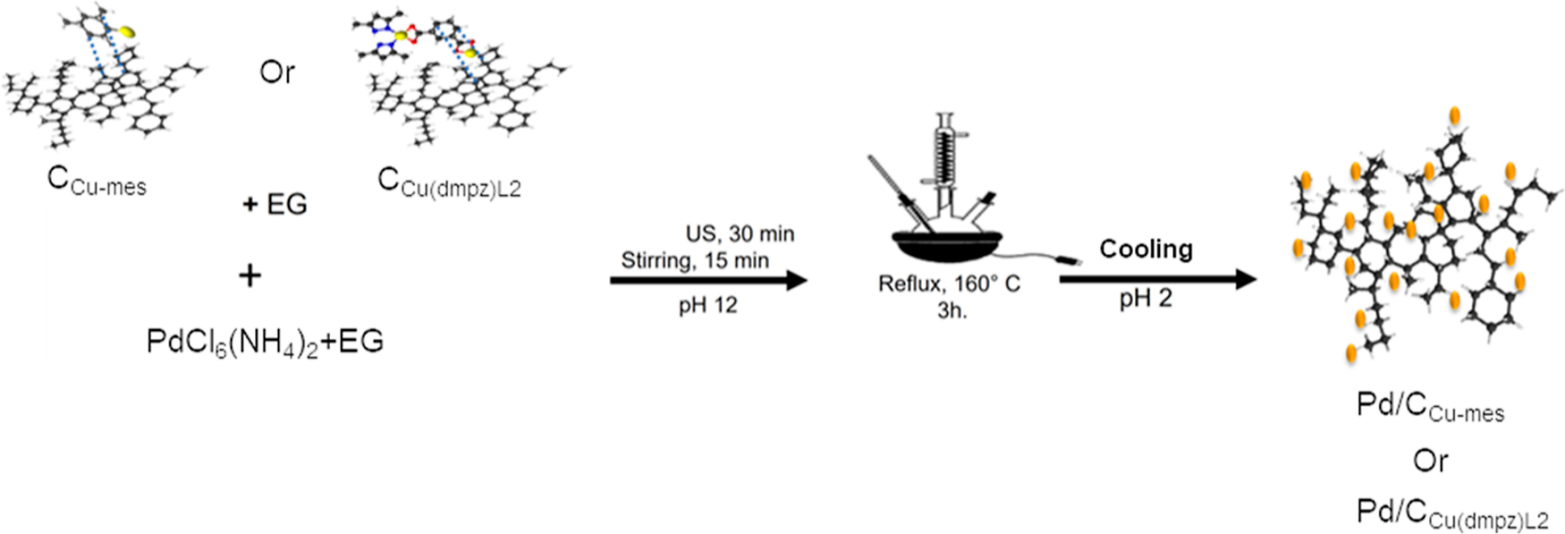
Synthesis of the Pd/C_Cu-mes_ and Pd/C_Cu(dmpz)L2_ Nanocatalysts

The 20 wt % Pd/C nanocatalyst was synthesized by the polyol method. C (0.08 g) was dispersed in 46 mL of EG, while 0.025 g of PdCl_6_(NH_4_)_2_ were separately mixed in 2 mL of ethanol. The dispersions were sonicated for 1 h. Afterward, the solution containing Pd was added dropwise to that of C and then stirred for 30 min in ambient conditions. The pH was adjusted to 12 by adding NaOH (1 mol L^–1^), the temperature increased to 160 °C and maintained for 3 h under refluxing and stirring conditions. The mixture was allowed to cool to room temperature, and 1 mol L^–1^ H_2_SO_4_ was added to adjust the pH to 2. Finally, the product was filtered, washed, and dried. The 20 wt % Pd/C_Cu-mes_ and Pd/C_Cu(dmpz)L2_ nanocatalysts were obtained following the same procedure. Scheme 3 shows the synthesis procedure to obtain Pd/C_Cu-mes_ and Pd/C_Cu(dmpz)L2_.

### 2.5 Physicochemical Characterization

FT-IR spectra were obtained in transmission mode in a WQF-S10A FT-IR Rayleigh instrument, at 4 cm^–1^ resolution in the scan range of 4000–500 cm^–1^. Raman microanalyses were carried out using a DXRZ Thermo Scientific equipment (λ = 633 nm, range between 400 and 3500 cm^–1^). X-ray diffraction (XRD) patterns were acquired in a Philips X’Pert diffractometer, with CuKα radiation (λ = 1.5418 Å). The lattice parameter (*a*_fcc_) was calculated with Bragg’s law using data from the Pd(111) peak
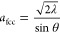
1where λ is the wavelength of the radiation emitted by the Cu K α lamp (1.5418 Å) and θ is the angle at the peak maximum. The fraction of Cu alloyed (*D*) in the nanocatalysts was calculated with eq 2
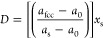
2where *a*_0_ is the experimental lattice parameter of the synthesized Pd/C, *a*_s_ is the lattice parameter of ca. 100% alloyed Pd–Cu/C, and *x*_s_ is the atomic fraction of Cu in the nanocatalysts.

The crystallite size (*d*_XRD_) was estimated using values of the (220) Pd plane with the aid of the Scherrer Equation
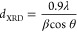
3where λ and θ have the same meaning as in eq 1, β is the full width at half-maximum in radians, and 0.9 is the Scherrer constant.

The chemical composition was determined in a Philips XL30 Scanning Electron Microscope, equipped with an EDS detector under an accelerating voltage of 20 kV. Characterization by HRTEM was performed in a Talos F200X microscope at an accelerating voltage of 200 kV.

XPS spectra were obtained in a Thermo Scientific Escalab 25OXi (source: Al–Kα, 1486.68 eV) spectrometer. Deconvolutions of the spectra were performed by using the Shirley-Sherwood baseline correction method. The binding energies (BEs) were calibrated to 284.8 eV due to adventitious carbon.

The d-band center (*E*) of the nanocatalyst was determined by using the valence band (VB) spectra obtained by XPS. A Shirley-type background correction was applied. To ensure a precise comparison of all VB, the upper limit of integration for background subtraction was fixed at a BE of 10.0 eV. The maximum value of the VB (VBM) was used as a reference point to obtain the energy of the Fermi level (*E*_F_). The VBM was determined by extrapolating the valence spectra edge. The difference between the d-band center and the Fermi level (*E* – *E*_F_) is crucial for determining the adsorption strength of the nanocatalysts.

The area under the curve of the valence spectra was integrated by using the trapezoidal method. Then, *E* related to *E*_F_ was calculated using a weighted sum of the energies of each state in the valence spectra, known as the centroid,^25,26^ which was determined in Matlab with the following equation
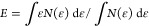
4where ε is the energy of a given state in the VB and *N*(ε) is the density of states at that energy level.

### 2.6 Electrochemical Half-Cell Characterization

Measurements were carried out in a three-electrode cell setup at room temperature in a Pine Wave Driver 20 bipotentiostat. The counter-electrode was a Pt wire in a separate compartment, while the reference electrode was of the Ag/AgCl type in saturated 3 M NaCl solution placed in a Luggin capillary, both with a membrane at the tip. The potentials were referred to as the reversible hydrogen electrode (RHE). The catalytic inks were prepared by separately mixing 10 mg of each nanocatalyst, 5 μL of Nafion, and 1 mL of 2-propanol by sonification for 40 min. The working electrodes were fabricated by transferring an aliquot of 10 μL of the catalytic ink into a glassy carbon disk (geometric area of 0.196 cm^2^).

Cyclic voltammograms (CVs) in a N_2_ atmosphere were obtained in a potential window between 0.05 and 1.2 V/RHE at a sweep rate of 20 mV s^–1^.

CO-stripping measurements were carried out by bubbling CO into the cell for 10 min while polarizing the electrode at 50 mV/RHE, followed by purging with Ar for 30 min. Then, two CVs were recorded at 20 mV s^–1^ in the range of 0.05 to 1.2 V/RHE, detecting the CO desorption (CO_des_) peak in the first cycle. ECSA values from CO-stripping (ECSA_CO_) were obtained from the integration of the experimental charge associated with the CO_des_ peak, according to Equation
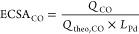
5where *Q*_CO_ (μC) is the charge due to the oxidation of CO_des_, *Q*_theo,CO_ is the theoretical charge for the oxidation of a monolayer of CO on a Pd electrode (420 μC cm^–2^), and *L*_Pd_ (μg) is the Pd loading.

Moreover, ECSA values from the CVs of the nanocatalysts (ECSA_PdO_) were estimated with equation
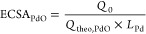
6where *Q*_0_ (μC) is the Coulombic charge from the peak due to the reduction of PdO species, *Q*_theo,PdO_ is the theoretical charge of 405 μC cm^–2^ needed for the reduction of a monolayer of Pd-oxides,^27^ and *L*_Pd_ (μg) has the same meaning as in eq 5.

Polarization curves of the EOR were acquired by adding a solution of 0.5 mol L^–1^ of C_2_H_5_OH to the 0.5 mol L^–1^ of KOH electrolyte in the same potential interval and a sweep rate of 20 mV s^–1^. The current of the reaction mixture was normalized with respect to the experimental Pd mass content to obtain the mass current density (*j*_m_). Accelerated degradation tests (ADTs) were performed by submitting the nanocatalysts to 2000 cycles at 200 mV s^–1^ in the 0.05–1.2 V/RHE potential interval.

### 2.7 Anion Exchange Membrane Direct Ethanol Fuel Cell

The performance of the nanocatalysts was studied in a homemade ethanol/O_2_ Teflon cell, using a PGSTAT302N Autolab potentiostat/galvanostat at room temperature. It must be highlighted that the cell setup served the purpose of comparing the performance of the nanocatalysts, but it was by no means intended to be a commercial-type AEM-DEFC.

The cell design included two Teflon compartments separated by a Fumatech Fumasep FAA AEM. The electrolytes were 0.5 mol L^–1^ KOH in the cathode and 0.5 mol L^–1^ ethanol +0.5 mol L^–1^ KOH in the anode compartments. N_2_ and O_2_ were bubbled, respectively.

To fabricate the anodes and cathodes used in the AEM-DEFC, conductive Toray carbon paper (2050-L FuelCell Store) was used as the support electrode. Pieces having a 5 × 5 mm area were cut, at which the nanocatalysts were deposited. The catalytic ink was prepared by mixing 2 mg of nanocatalyst with 95 μL of 2-propanol and 5 μL of Nafion. The metal loading on each anode and cathode was 0.20 mg_metal_ cm^–2^. In the configuration proposed, anodes and cathodes contained the same nanocatalyst.

The method used to characterize the performance of the cell was chronopotentiometry. It consisted of applying a controlled current (*i*) between the anode and cathode in a galvanostatic mode. The anode acting as counter electrode and a Basi reference electrode were placed in the same chamber during measurements, while the working electrode was the cathode. Thus, the *i* imposed was negative (−50 μA to −1 mA). The performance of the nanocatalysts was compared by obtaining independent polarization curves of the anode (*E*_a_) and cathode (*E*_c_) potentials as a function of current density (*j*).

The measured potential difference between the anode and cathode was the fuel cell voltage (*E*_cell_).

The fuel cell power density (*P*_cell_) was determined using Ohḿs relationship
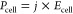
7

*E*_cell_ vs *j* and *P*_cell_ vs *j* curves were plotted and shown.

### 2.8 Analysis of Reaction Products by High-Performance Liquid Chromatography

The species produced from the ethanol oxidation were quantified by high-performance liquid chromatography (HPLC) after electrolysis for 4 h at an applied potential of 0.8 V/RHE. The configuration of the AEM-DEFC changed slightly compared with that described above. The working electrodes were the nanocatalysts evaluated as anodes with the reference electrode placed in the same compartment. Meanwhile, an Fe/N-rGO cathode in the second chamber was the counter electrode.

Using this setup, it was possible to obtain the percentage of ethanol consumed as well as the percentage of the subproducts due to its oxidation. The subproducts were separated in a Biorad HPLC organic acid analysis column (Animex HPX-87) operating under isocratic conditions. The carrier consisted of 3.33 mmol L^–1^ H_2_SO_4_ at a flow rate of 0.6 mL min^–1^ using He (99%, alphagaz). Aliquots were collected each hour and injected into a Thermo Scientific Dionex Ultimated 3000 UHPLC. The reaction products were quantitatively determined by comparing their retention times with those of pure commercial standards injected under the same analysis conditions.

## 3 Results and Discussion

### 3.1 Physicochemical Characterization of Vulcan Supports and Pd Nanocatalysts

**Figure 1:**
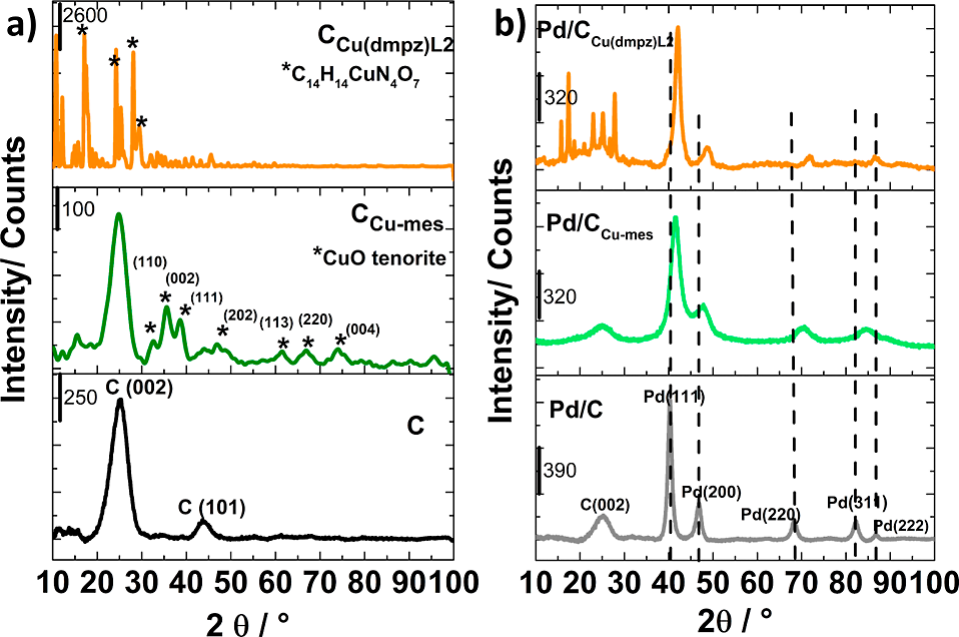
XRD patterns of (a) C, C_Cu-mes_, and C_Cu(dmpz)L2_; (b) Pd/C, Pd/C_Cu-mes_, and Pd/C_Cu(dmpz)L2_.

The XRD patterns of C, C_Cu-mes_, and C_Cu(dmpz)L2_ are shown in Figure 1a. C displays peaks around 2θ = 25.40 and 43.54°, due to the (002) and (101) planes of graphite (JCPDS 74-2329), confirming that Vulcan has both amorphous and graphitic features.^28^

Besides the (002) reflection, C_Cu-mes_ shows reflections at 32.57, 35.76, 38.97, 47.05, 61.03, 67.41, and 74.13° (2θ), corresponding to the (110), (002), (111), (202), (113), (220), and (004) planes of tenorite (CuO, JCPDS 41–0254).^29,30^ Meanwhile, C_Cu(dmpz)L2_ shows high-intensity reflections ascribed to C_14_H_14_CuN_4_O_7_, which hinder those of graphite. These features evidently show the effect of functionalizing C with the organometallic compounds on its structural characteristics. This confirms the correct coordination of the Cu compounds on the carbon surface. The FT-IR, Raman, and XPS spectra, as well as the chemical composition of the three carbon supports, are shown and discussed in Section S1 of the Supporting Information file. Moreover, part of the physicochemical and electrochemical characterization of the Pd nanocatalysts is shown and discussed in Section S2.

Figure 1b shows the XRD patterns of the nanocatalysts. The reflection close to 2θ = 25° is ascribed to the (002) plane of the graphitic carbon structure. In Pd/C, the peaks located at 40.3, 46.9, 68.2, 82.1, and 86.8° (2θ) are attributed to the (111), (200), (220), (311), and (222) planes of crystalline fcc Pd (JCPDS 46-1043).^31,32^

In the case of the nanocatalysts supported on functionalized carbon, the Pd planes shift toward higher degrees (2θ) suggesting a modification of the Pd lattice due to the formation of fcc PdCu alloyed phases (JCPDS 48-1551).^33,34^ There are no additional peaks related to Cu^0^ or Cu oxides. Nevertheless, their presence should not be discarded.^35^ The displacement of the Pd planes is more evident in Pd/C_Cu(dmpz)L2_ and this is mainly attributed to the high Cu content exposed at this nanocatalyst (Table S4). Moreover, it shows several peaks in the 15–25° (2θ) interval attributed to the organometallic compound, as observed in the diffractogram of C_Cu(dmpz)L2_ in Figure 1a.

Thus, the shift of the reflections toward higher 2θ degrees in Figure 1b is attributed to the formation of PdCu alloyed phases. To confirm this, using data from the (111) Pd plane, the lattice parameter (*a*_fcc_) and the fraction of Cu alloyed (D) have been calculated and are depicted in Table S5. Pd/C_Cu-mes_ and Pd/C_Cu(dmpz)L2_ have an obvious lattice contraction compared to Pd/C, resulting in *a*_fcc_ = 0.375 and 0.369 nm, respectively. This is evidence that Cu atoms from the organometallic compounds and Pd nanoparticles promote the formation of PdCu alloyed phases on both nanocatalysts.

Additionally, the D values of Pd/C_Cu-mes_ and Pd/C_Cu(dmpz)L2_ are 31 and 33%, respectively. These *a*_fcc_ and D values are similar to those reported in the literature for conventional PdCu alloys.^36−38^ Thus, strong evidence of the formation of the PdCu alloy at Pd/C_Cu-mes_ and Pd/C_Cu(dmpz)L2_ is obtained from this characterization.

Table S5 summarizes the mean crystallite size values (*d*_XRD_) of the nanocatalysts from the information on the Pd(220) reflection. Pd/C_Cu-mes_ has the smallest *d*_XRD_ compared to Pd/C_Cu(dmpz)L2_ and Pd/C. The small *d*_XRD_ value of Pd/C_Cu-mes_ can be correlated to the presence of nucleation centers (surface functional groups and Cu sites), which limit the particle size growth due to the functionalization with Cu-mes. Pd/C_Cu(dmpz)L2_ has also a smaller *d*_XRD_ value than Pd/C. It should be mentioned that high dispersion and small particle size play a key role in the catalytic activity since these parameters have a critical effect on the ECSA.^32^

**Figure 2:**
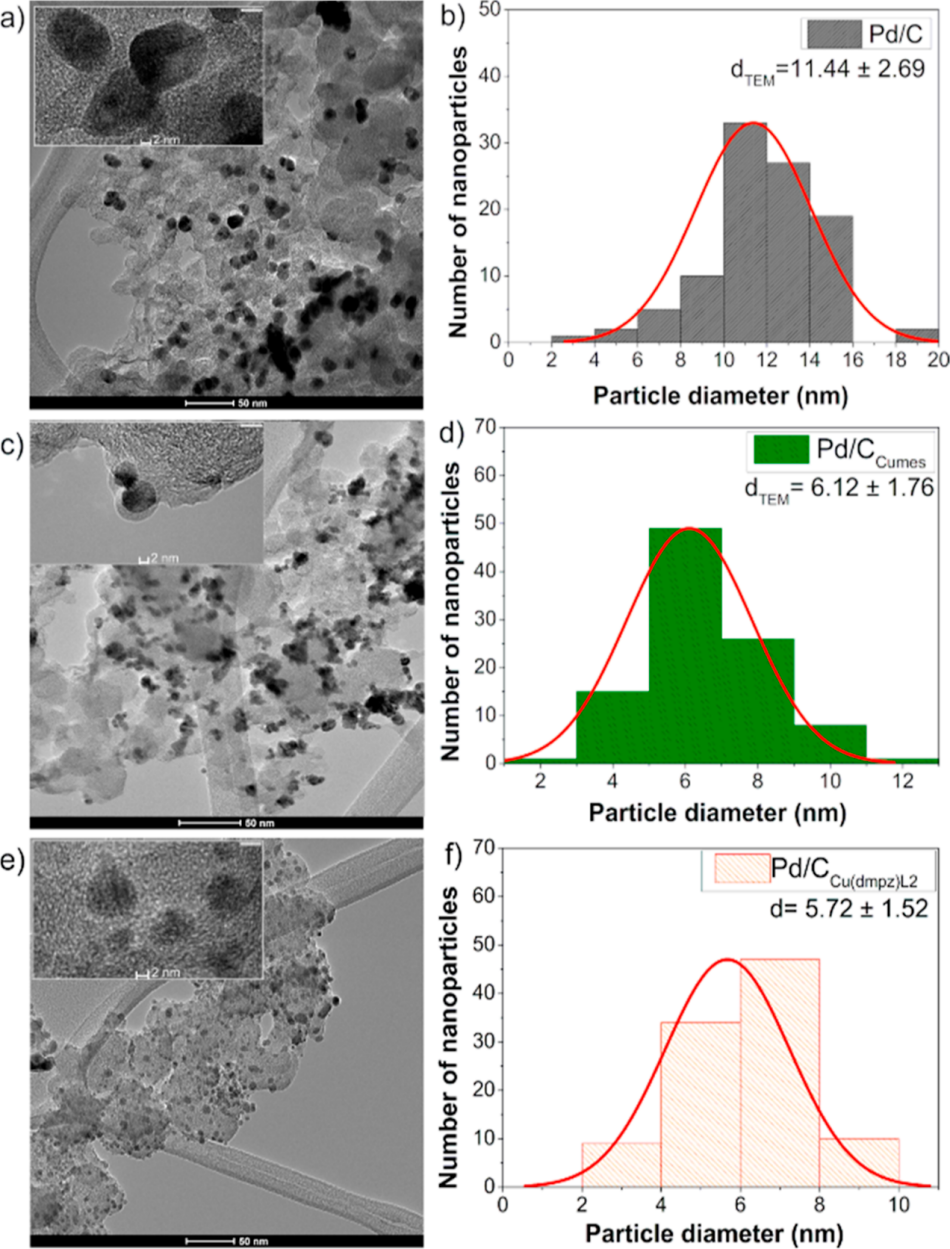
TEM micrographs and corresponding particle size (*d*_TEM_) histograms of Pd/C (a,b), Pd/C_Cu-mes_ (c,d), and Pd/C_Cu(dmpz)L2_ (e,f). Insets: HRTEM images of representative nanoparticles.

Figure 2a shows TEM micrographs of the Pd/C nanocatalyst. Dispersed dark nanoparticles on the support are attributed to Pd. The inset is an HRTEM image of the nanoparticles. The histogram in Figure 2b shows the resulting particle size distribution of the nanocatalyst with an average *d*_TEM_ = 11.44 ± 2.69 nm (Table S5). In the case of Pd/C_Cu-mes_ (Figure 2c), agglomeration is observed, although the micrograph shows also some dispersed nanoparticles (*d*_TEM_ = 6.12 ± 1.76, Figure 2d and Table S5). Figure 2e,f shows micrograph and histogram, respectively, of Pd/C_Cu(dmpz)L2_. It has a better dispersion compared to the other two nanocatalysts, having *d*_TEM_ = 5.72 ± 1.52 nm. It should be noted that Pd/C_Cu(dmpz)L2_ has a higher Cu concentration and *D* value than Pd/C_Cu-mes_ (Tables S4 and S5), so it is inferred that Cu-species positively influence the anchorage and homogeneous dispersion of Pd nanoparticles. Therefore, the TEM results show with more clarity the positive effect of the functionalization of Vulcan on limiting the Pd particle size growth.

The high angle annular dark field images and chemical mapping of Pd/C, Pd/C_Cu-mes_, and Pd/C_Cu(dmpz)L2_, along with their Raman spectra are shown and discussed in Figures S4–S7 of Section S2.

**Figure 3:**
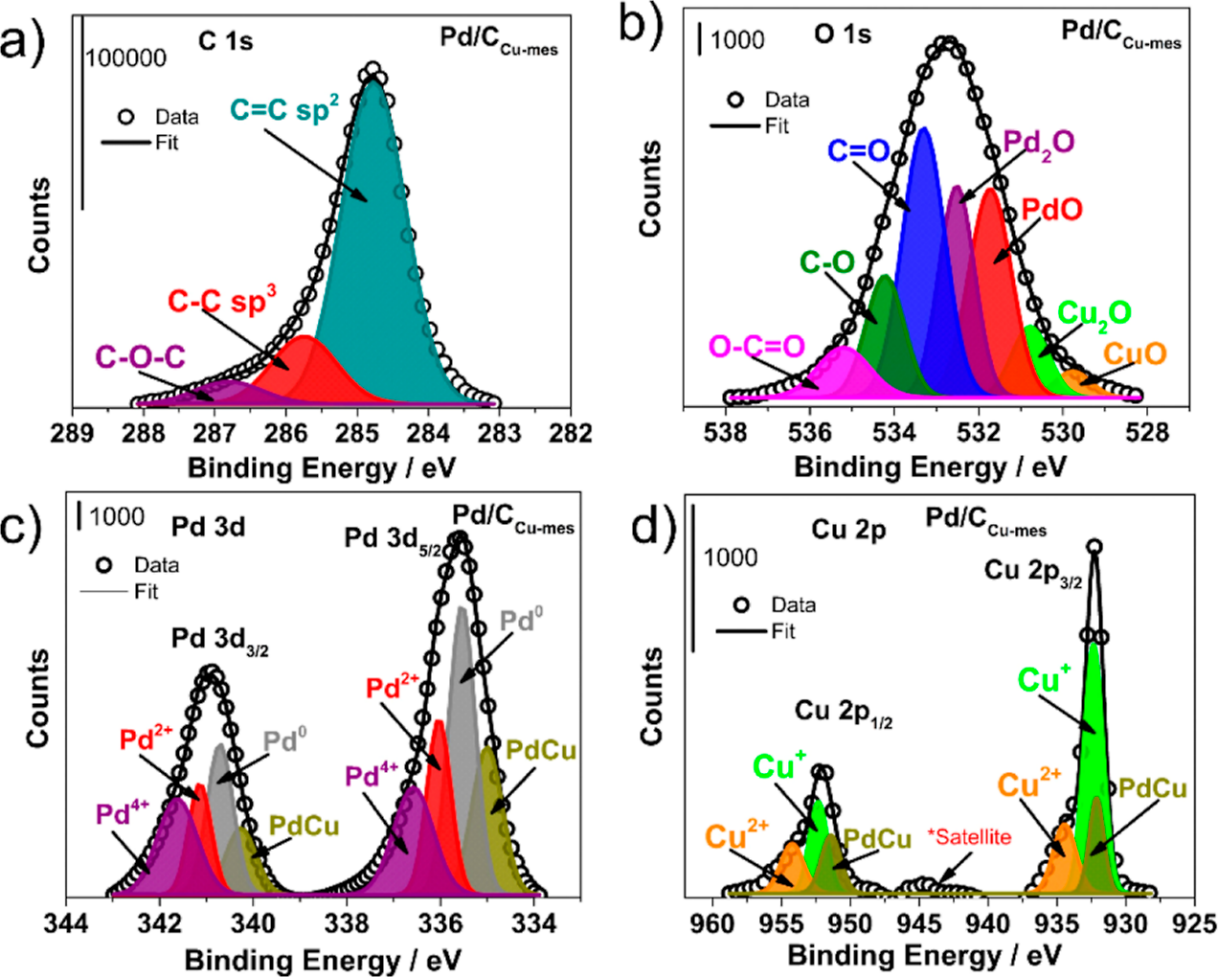
XPS spectra of Pd/C_Cu-mes_ in the (a) C 1s, (b) O 1s, (c) Pd 3d, and (d) Cu 2p regions.

The XPS spectra in Figure 3 show the (a) C 1s, (b) O 1s, (c) Pd 3d, and (d) Cu 2p regions of Pd/C_Cu-mes_. The C 1s region has the most intense signal at 284.77 eV assigned to sp^2^ hybridizations (C=C bonds) with a relative concentration of 77.5 at. % (Table S7), followed by the sp^3^ hybridizations (C–C bonds, BE = 285.73 eV) and the C–O–C species (BE = 288.77 eV). Meanwhile, the region of the O 1s has been deconvoluted into seven peaks. The signals at 529.94 and 530.82 eV correspond to CuO and Cu_2_O bonds, respectively. These species have lower relative concentrations than the other species detected (Table S7). However, their identification confirms the presence of the Cu species bonding with O atoms.

Additionally, the signals assigned to PdO and PdO_2_ are displayed at 531.72 and 532.52 eV, which confirms the bonding interaction between Pd and O (see their relative concentration in Table S7). The other peaks correspond to C=O, C–O, and C–C=O bonds ascribed to the functional groups from the support. At the Pd 3d region, four doublets from the spin–orbit splitting into the Pd 3d_5/2_ and Pd 3d_3/2_ states confirm the formation of the PdCu phase (BE = 335.0 and 340.26 eV), metallic Pd (Pd^0^, BE = 335.54 and 340.71 eV), PdO (Pd^2+^, BE = 336.03 and 341.14 eV), and PdO_2_ (Pd^4+^, BE = 336.58 and 341.62 eV). The higher relative concentration corresponds to Pd^0^ (35.3 at %), followed by the Pd^4+^, Pd^2+^, and PdCu species (Table S7). The deconvoluted peaks in Figure 3 have estimated full width at half maximum (fwhm, Table S7) values close to those reported in the literature.^39−41^

It is important to note that the Pd doublets in Pd/C_Cu-mes_ show a shift toward higher BE compared to the Pd/C nanocatalyst of which the XPS spectra have been reported in ref (42). Specifically, Pd^0^ in the Pd 3d_5/2_ state is centered in 335.54 eV at Pd/C_Cu-mes_, a shift of 0.4 eV compared to 335.14 eV of the same species at Pd/C. The displacement is attributed to a modification of the d-band of Pd due to an electron transfer from Cu atoms which increases the BE of the 3d electrons of Pd, shifting their peaks implying the formation of PdCu alloyed phases, and providing strong evidence of alloy structure and composition as reported elsewhere,^43,44^ in good agreement with the observations from XRD and HRTEM analyses.

The Cu 2p spectra are also deconvoluted into three doublets at the spin–orbit splitting of Cu 2p_3/2_ and Cu 2p_1/2_. The peaks at around 932.13 and 951.41 eV emerge from PdCu phases, with a relative concentration of 21.8 atom % (Table S7). Meanwhile, the doublets at 932.35 and 952.33 eV are due to Cu^+^ species (with the highest relative concentration: 54.9 at %), and those at 934.45 and 954.22 eV are attributed to Cu^2+^ species (23.3 at %, Table S7). In addition, the presence of a satellite peak at BE = 945 eV confirms the formation of Cu oxides, as discussed in Figure 5.

**Figure 4:**
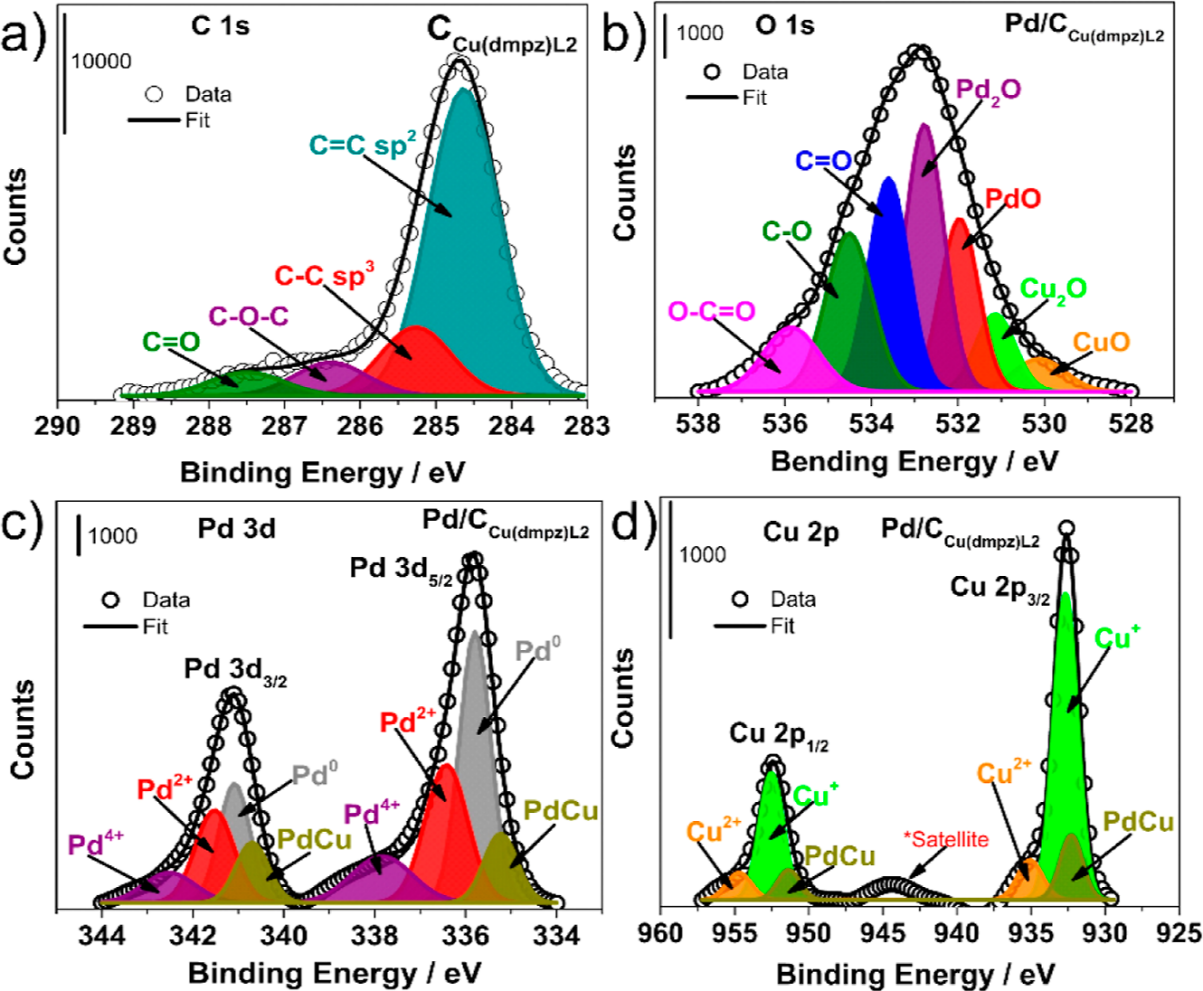
XPS spectra of Pd/C_Cu(dmpz)L2_ in the (a) C 1s, (b) O 1s, (c) Pd 3d, and (d) Cu 2p regions.

Figure 4 shows the XPS spectra of Pd/C_Cu(dmpz)L2_ in the (a) C 1s, (b) O 1s, (c) Pd 3d, and (d) Cu 2p regions. In the C 1s region, besides the C=C (sp^2^ hybridization), C–C (sp^3^ hybridization), and C–O–C as in the case of Pd/C_Cu-mes_ (in Figure 3a), the C=O (BE = 287.76 eV), and O–C=O (BE = 289.50 eV) bonds also emerge. This is an evident effect of the different chemical structures of Cu(dmpz)L2 compared to Cu-mes. Meanwhile, the O 1s region displays the peaks corresponding to CuO, Cu_2_O, PdO, and Pd_2_O, the latter with the highest relative concentration (25.5 at %, Table S7). Additionally, signals ascribed to C=O, C–O, and the C–C=O bonds are shown.

The deconvoluted spectra of the Pd 3d region in Pd/C_Cu(dmpz)L2_ display four doublets corresponding to PdCu bonds (BE = 335.23 and 340.70), Pd^0^ (BE = 335.74 and 341.08 eV), Pd^2+^ (BE = 336.72 and 341.96 eV), and Pd^4+^ (BE = 338.64 and 342.52 eV). Pd^0^ in the Pd 3d_5/2_ state exhibits a shift of 0.6 eV toward higher BE compared to Pd/C in.^42^ The shift is more important than that shown by Pd/C_Cu-mes_, indicating stronger interactions tending to form PdCu alloyed phases at Pd/C_Cu(dmpz)L2_. Even more, three doublets are observed in the Cu 2p_3/2_ and Cu 2p_1/2_ states, due to the Cu^+^, Cu^2+^, and PdCu species. Similar to the previous nanocatalyst, the highest relative concentration is that of Cu^+^ (72.6 at %, Table S7).

### 3.2 Electrochemical Behavior of the Nanocatalysts

**Figure 5:**
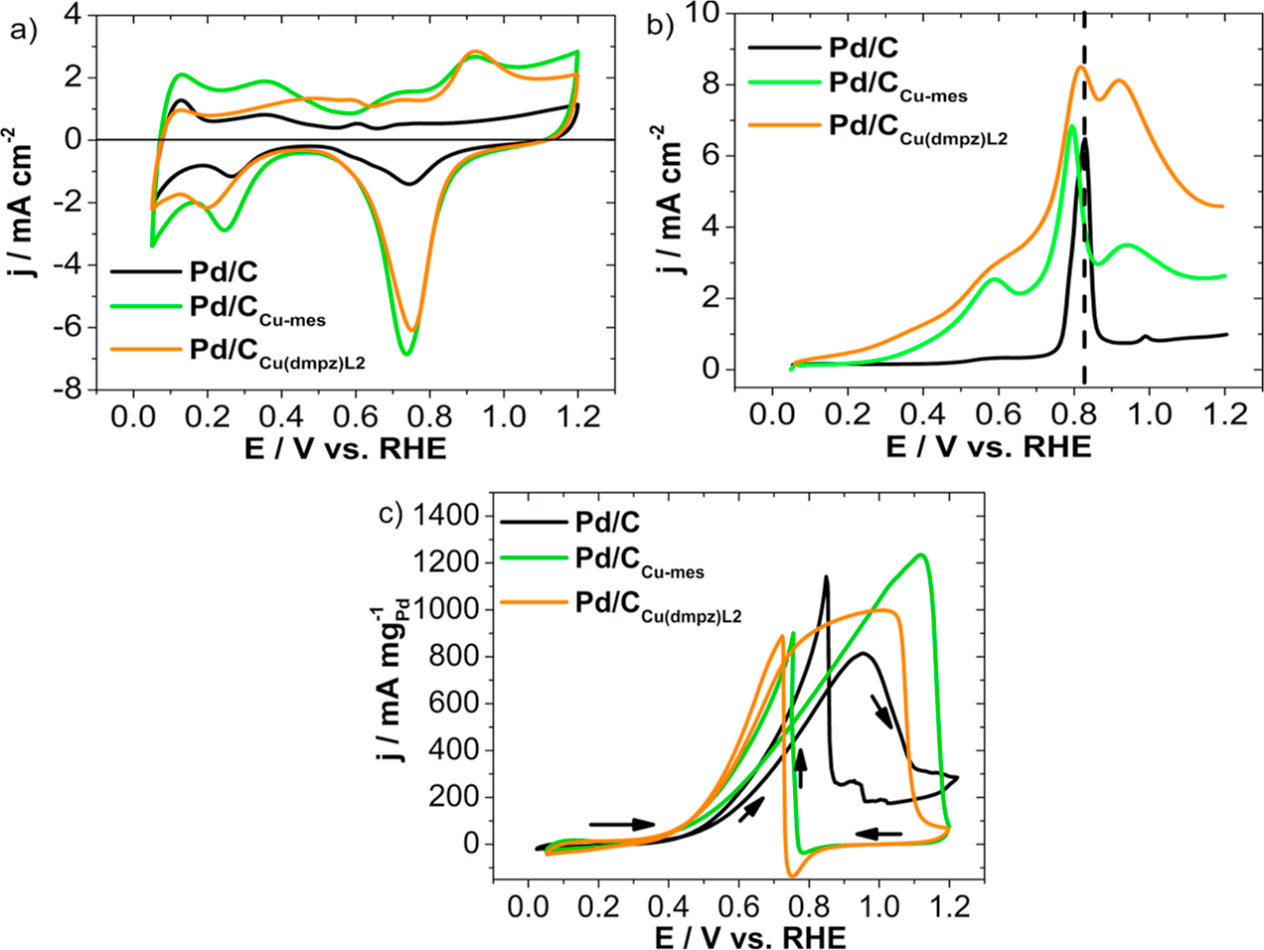
(a) CVs of Pd/C, Pd/C_Cu-mes_, and Pd/C_Cu(dmpz)L2_ in N_2_-saturated 0.5 mol L^–1^ KOH recorded at 20 mV s^–1^. (b) CO-stripping curves at the nanocatalysts in 0.5 mol L^–1^ KOH saturated with N_2_ recorded at 20 mV s^–1^. (c) Polarization curves at Pd/C, Pd/C_Cu-mes_, and Pd/C_Cu(dmpz)L2_ in 0.5 KOH mol L^–1^ in the presence of 0.5 mol L^–1^ EtOH recorded at 20 mV s^–1^.

Figure 5a shows the CVs of the nanocatalysts. The characteristic peaks related to Pd-based materials are shown, i.e., the hydrogen adsorption/desorption (*H*_ads/des_) region in the 0.05–0.40 V/RHE interval, the double layer region between ∼0.50 and 0.60 V/RHE, and the Pd oxide formation/reduction between 0.60 and 1.2 V/RHE. The nanocatalysts supported on functionalized C show a higher current density (*j*) over the potential scanned, particularly a remarkable increase in the peak ascribed to the reduction of Pd oxides, compared to Pd/C.

Pd/C_Cu-mes_ and Pd/C_Cu(dmpz)L2_ show *j* peaks at 0.65 and 0.90 V/RHE in the anodic scan. The latter also shows a broad shoulder at ca. 0.50 V/RHE. Morais et al. attribute these features to redox properties of Cu-based sites, also suggesting that the reactions that occur in the formation of the prepeak at 0.65 V/RHE are^43^
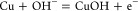
8
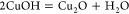
9while the peak at ca. 0.9 V/RHE is mainly due to the reactions
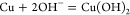
10
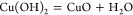
11

Cu_2_O and CuO as subproducts are insoluble species, probably leading to the passivation of Cu atoms at the surface of the nanocatalyst. The j peaks between 0.8 and 0.7 V/RHE in the backward scan can be attributed to the reduction of the Pd oxides, as well as to the reprecipitation of Cu from soluble Cu^+^ and Cu^2+^ species,^45−47^ as well as the presence of those Cu species, which are confirmed from XPS analysis.

Figure 5b depicts the CO-stripping curves at the developed nanocatalysts. Pd/C shows CO stripping with an onset potential (*E*_onset,CO_) of 0.70 V/RHE and an oxidation potential (*E*_ox_) of 0.82 V/RHE (Table S8). This *E*_ox_ value aligns closely with the value reported elsewhere for commercial Pd/C electrocatalyst (0.879 V/RHE).^47^ Meanwhile, the Pd/C_Cu-mes_ nanocatalyst has a remarkable shift of both potentials to more negative values (*E*_onset,CO_ = 0.19 and *E*_ox_ = 0.79 V/RHE), suggesting that poisoning CO species can be removed more easily. Meanwhile, Pd/C_Cu(dmpz)L2_ also shows an enhanced effect, with values of *E*_onset,CO_ = 0.13 and *E*_ox_ = 0.81 V/RHE.

It is interesting to observe that Pd/C features a single peak during CO oxidation, whereas Pd/C_Cu-mes_ develops a prepeak at 0.59 V/RHE, in addition to the previously mentioned peak at 0.79 V/RHE. Moreover, the *j* peak at 0.92 V/RHE most likely corresponds to Cu-species as described by reactions (10) and (11) and can be correlated to the peaks in Figure 5a. Pd/C_Cu(dmpz)L2_ also shows a prepeak, a CO-oxidation peak, and a third peak due to the Cu species (0.58, 0.81, and 0.90 V/RHE, respectively).

It has been reported that the oxidation of adsorbed CO species (CO_ads_) at more negative potentials is promoted by the bifunctional mechanism and the electronic effect.^48^ In the former, Cu-atoms form surface OH^–^ species at more negative potentials which are transported to Pd sites, promoting an easier oxidation of CO_ads_. Moreover, the formation of PdCu alloyed phases leads to a modification of the electronic structure of Pd, weakening the adsorption energy of CO_ads_ species, facilitating their oxidation.^49^

The broadness and the presence of multiple CO-stripping peaks at Pd/C_Cu-mes_ and Pd/C_Cu(dmpz)L2_ is attributed to the oxidation of weak and strong CO_ads_ at different Pd planes, including the ones at 0.79–0.81 V/RHE. This effect is reported elsewhere and has been attributed it to a modification in the CO adsorption mechanism on the surface of the Pd sites.^50^ Another reason for such a feature is that Pd/C_Cu-mes_ and Pd/C_Cu(dmpz)L2_ contain unalloyed Pd (as seen from XPS analysis, Figures 3 and 4) which provokes the desorption of CO_ads_ at more negative potentials than in the case of Pd/C. Actually, it has been reported that the ECSA value has some degree of relationship with the percentage of Pd^0^ in Pd-based catalysts, which is intrinsically related to the alloying degree.^51^ In contrast, Pd/C seems to oxidize only one strongly adsorbed CO_ads_ species at more positive potentials.

The ECSA from CO-stripping (ECSA_CO_) has been determined from the charge due to the CO oxidation peak in Figure 5b using eq 5, obtaining 46.0, 102.8, and 145.2 m^2^ g^–1^ for Pd/C, Pd/C_Cu-mes_, and Pd/C_Cu(dmpz)L2_, respectively (Table S8). The ECSA_CO_ of Pd/C is similar to that reported in the literature.^52^ Nevertheless, those of Pd/C_Cu-mes_ and Pd/C_Cu(dmpz)L2_ are higher probably due to the presence of Cu species, which promote some active sites with high activity for CO oxidation. As a result, the synergistic effect between Pd and Cu promotes a higher catalytic activity in terms of *E*_onset,CO_ and *E*_ox_ compared to Pd/C.

The polarization curves of the EOR at the Pd/C, Pd/C_Cu-mes_, and Pd/C_Cu(dmpz)L2_ nanocatalysts are shown in Figure 5c. The nanocatalysts display a typical behavior for the reaction, except for Pd/C which shows two peaks in the backward scan, the same that are discussed in detail in ref (42). At the more negative potentials, ethanol adsorption proceeds on the surface-active sites at the nanocatalyst, followed by its dissociation. Such surface reactions hinder the *H*_ads/des_ region seen in Figure 5a.

The Pd/C_Cu-mes_ and Pd/C_Cu(dmpz)L2_ nanocatalysts promote the EOR at a more negative *E*_onset_ (0.38 V/RHE) compared to Pd/C (0.41 V/RHE) as seen in Table S8, due to a modification of the Pd lattice because of PdCu alloying. Moreover, the mass current density (*j*_m_) peak is considerably higher at Pd/C_Cu-mes_ (1231.3 mA mg_Pd_^–1^), i.e., 1.2 and 1.5 times more intense than those of Pd/C_Cu(dmpz)L2_ and Pd/C, respectively (Table S8), showing that the electronic effect is very influential at this nanocatalysts by modifying the energy of adsorption of ethanol and intermediates. It can be mentioned, however, that the *j*_m_ of Pd/C (808.3 mA mg_Pd_^–1^) is higher than those reported previously.^47,53^

Nevertheless, it should be highlighted that Pd/C_Cu(dmpz)L2_ delivers higher *j*_m_ values in the potential ranging from ca. 0.45 to 0.9 V/RHE than Pd/C_Cu-mes_, and is more important than Pd/C over the full scan. The curve at Pd/C_Cu(dmpz)L2_ is broader, indicating the oxidation of ethanol and intermediate species over a wider, more negative potential interval. This electrocatalytic behavior shows that the electronic effect and the bifunctional mechanism are highly relevant in this nanocatalyst.

Evidently, the nanocatalysts supported on carbon functionalized with Cu organometallic compounds have enhanced performance for the reaction. Such high catalytic activity can be related to the high ECSA from the reduction of Pd-oxides (ECSA_PdO_) of Pd/C_Cu-mes_ and Pd/C_Cu(dmpz)L2_ obtained with Equation 6, i.e., 118.7 and 148.0 m^2^ g^–1^, compared to 29.4 m^2^ g^–1^ of Pd/C before ADT (Table S8), attributed to a large number of active sites participating in the reaction, including those of Cu oxides.

The improvement in catalytic activity for the EOR of Pd/C_Cu-mes_ and Pd/C_Cu(dmpz)L2_ (in terms of *E*_onset_ as well as the shift of current densities toward more negative potentials) compared to Pd/C, can be ascribed in part to an easier removal of CH_3_CO_ads_ (Figure 5b). The latter is mainly attributed to the bifunctional mechanism, which can be explained according to the following reactions
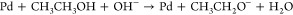
12
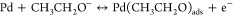
13
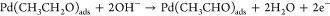
14
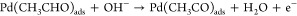
15
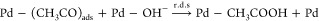
16
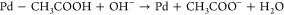
17
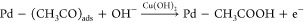
18

The main product of ethanol electrooxidation is acetate, due to either incomplete electrooxidation or a multistep reaction. As noted above, the reactive intermediates such as CH_3_CH_2_O_ads_ and CH_3_CO_ads_ are adsorbed on the surface of Pd, blocking active sites (reactions 12–15). This behavior generally occurs at negative potentials in the anodic sweep. At more positive potentials, the production of OH^–^ species is promoted on the Pd surface, displacing the strongly adsorbed carbonaceous species (reaction 16) and ultimately increasing the *j* generated. In the presence of Cu, the OH^–^ groups are generated at more negative potentials promoting the release of strongly adsorbed intermediates on Pd active sites and then enhancing the rate of complete ethanol oxidation (reactions 17 and 18).^54^

Even more, the presence of Cu^2+^ species favors the formation of OH^–^ species, which modifies the EOR pathway where acetaldehyde is oxidized to acetic acid, according to reactions^55^
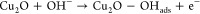
19
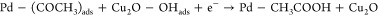
20

Therefore, the results also indicate an improvement in catalytic activity of Pd/C_Cu-mes_ and Pd/C_Cu(dmpz)L2_ promoted by a mechanism of the EOR following a path that produces acetic acid due to the presence of Cu-species.^55^ The same Cu-species have been reported to prompt a faster electron transfer rate,^47^ thus enhancing even more the catalytic performance of the nanocatalysts. It is likely also that upon the development of such reactions, the long-term stability of the nanocatalysts is improved by removing adsorbed poisoning intermediates that may limit or even decrease their performance.

Moreover, the improved j_m_ at Pd/C_Cu-mes_ and Pd/C_Cu(dmpz)L2_ is related to a modification of the electronic structure of Pd (electronic effect) because of the formation of PdCu alloyed phases. As a result, the adsorption energy of species such as ethanol and/or intermediates becomes weaker, facilitating bonds cleavages and thus promoting the generation of higher *j*_m_ values.^56,57^

According to several workers, there is a strong correlation between the d-band center of a nanocatalyst and the adsorption energy of species and reaction intermediates, which affects its catalytic activity and electrochemical stability.^36,58,59^ Hammer and Nørskov have proposed that the higher the energy of the d states compared to the Fermi level (*E*_F_), the greater the energy of the antibonding states and the greater the bond strength.^60^ Consequently, the energy difference between the d-band center (*E*) and *E*_F_ can be correlated to the adsorption energy of adsorbates on the active sites of the nanocatalyst.

Here, the d-band center of the nanocatalysts was calculated from the VB values determined from the high-resolution XPS spectra. Such centers can provide information about the energy interactions between the orbitals of the adsorbates and the d-orbitals of Pd.

**Figure 6:**
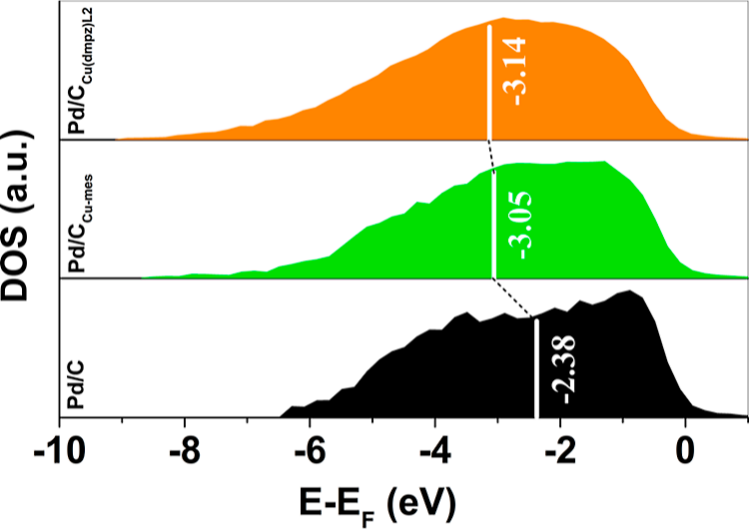
d-band center of Pd/C, Pd/C_Cu-mes_, and Pd/C_Cu(dmpz)L2_ determined from high-resolution XPS VBs.

Figure 6 shows the *E* – *E*_F_ values of the nanocatalysts. The *E* values of Pd/C_Cu-mes_ and Pd/C_Cu(dmpz)L2_ shift negatively compared to Pd/C, indicating a modification of the electronic structure of Pd due to the formation of PdCu alloyed phases. Therefore, there is an important effect of the functionalization of the carbon support on the d-band center of the nanocatalysts. First, there is a good correlation between the d-band center values with the lower catalytic activity of Pd/C for the EOR, compared to the two other anode materials. Second, the more negative d-band center of Pd/C_Cu(dmpz)L2_ is a strong indication of lower adsorption energies of adsorbates on its surface.^24^ Such behavior can explain the high performance and the important electronic effect shown by this nanocatalyst in Figure 5c.

Table S8 shows the electrochemical parameters of Pd/C after ADT from the results reported in ref (42). There is an increase in *j*_m_ after the test. Nevertheless, the ECSA_PdO_ loss of Pd/C is about 49% after ADT.

Figure S8 shows (a) CVs and (b) polarization curves of the EOR before and after ADT of Pd/C_Cu-mes._ Although the shape of the CV remains almost unchanged, it is remarkable to note the decrease of *j* over the potential scanned. Before ADT, Pd/C_Cu-mes_ shows an important ECSA_PdO_ value of 118.7 m^2^ g_Pd_^–1^ (Table S8), which is higher than those reported in the literature.^58,61,62^ After submission to ADT, the intensity of the peak due to the reduction of Pd-oxides decreases. The ECSA_PdO_ after ADT is 63.9 m^2^ g^–1^, i.e., a loss of ca. 46%. During the test, Pd/C_Cu-mes_ may undergo changes in the morphology and surface chemistry because of different degradation mechanisms. For instance, the dissolution of nanoparticles can occur. Also, the migration of Pd atoms is not unusual, resulting in the aggregation of nanoparticles, thus provoking ECSA_PdO_ losses.^62,63^

Comparing the polarization curves of the EOR at Pd/C_Cu-mes_ before and after ADT, the nanocatalyst shows a slight decrease of roughly 12% in *j*_m_, despite the relatively high ECSA_PdO_ losses (Table S8). These results suggest that most of the Pd and PdCu sites remain active to promote the reaction. The presence of Pd active sites correlates with the results obtained by XPS in which a higher concentration of Pd^0^ (35.5 atom %) is obtained compared to Pd-oxides. Therefore, it can be considered as an electrochemically stable anode nanocatalyst with a low decrease in *j*_m_ after being subjected to 2000 cycles in ADT.

Figure S9a shows the CVs before and after ADT of Pd/CCu(dmpz)L2. The same degradation mechanisms as described in the previous case may have caused the notable decrease in *j*_m_, clearly observed in the Pd-oxide reduction peak at this nanocatalyst as well as in the ECSAPdO losses (Table S8). The ECSAPdO after ADT of Pd/CCu(dmpz)L2 is 55.4 m^2^ g^–1^, i.e., a loss of about 62%. Considering the ECSAPdO losses after ADT, the stability of the nanocatalysts decreases in the order Pd/CCu-mes > Pd/C > Pd/CCu(dmpz)L2.

Figure S9b shows the polarization curves of the EOR on Pd/C_Cu(dmpz)L2_. There is a change in the shape of the curve after ADT, which becomes a relatively narrow peak. In contrast to Pd/C_Cu-mes_, Pd/C_Cu(dmpz)L2_ shows a slight increase in *j*_m_ by ∼8% (Table S8), suggesting that after ADT some sites, likely Pd-oxides that promote the oxidation of organic molecules, become activated^64^ correlating with the results obtained by XPS in which a percentage of 31.2 and 14.4 at % (PdO and Pd_2_O, respectively, Table S7) has been obtained. However, it should be acknowledged that the potential window at which species are oxidized is more limited after ADT, probably because some other sites (especially those that catalyzed the reaction at more negative potentials) are deactivated or degraded. The peak *j*_m_ of Pd/C_Cu(dmpz)L2_ is lower than that of Pd/C_Cu-mes_ after ADT, but the maximum is at a more negative potential. Thus, there is a relevant consequence of both the bifunctional mechanism and the electronic effect at this nanocatalyst, as seen in Figure 5c. Overall, the electrocatalytic performance of Pd/C_Cu-mes_ and Pd/C_Cu(dmpz)L2_ for the EOR, before and after ADT, overmatch that of Pd/C (Table S8).

Table S9 shows a comparison of electrochemical parameters of Pd/C_Cu-mes_ and Pd/C_Cu(dmpz)L2_ with some Pd- and Cu-containing nanocatalysts from elsewhere. As seen, the ECSA_PdO_ of Pd/C_Cu-mes_ and Pd/C_Cu(dmpz)L2_ is significantly higher. Regarding the EOR, the *E*_onset_ values of these nanocatalysts are among the more negative. As for the j peak (in mA cm^–2^ for comparison purposes), the nanocatalysts in this work (119.6 and 81.2 mA cm^–2^ at Pd/C_Cu-mes_ and Pd/C_Cu(dmpz)L2_, respectively) compare positively with those reported by other workers, particularly Pd/C_Cu-mes_.

In order to identify the products of the EOR at the nanocatalysts, an electrolysis test at 0.8 V/RHE have been performed. The percentage of ethanol consumed is 47, 61, and 59% at Pd/C, Pd/C_Cu-mes_, and Pd/C_Cu(dmpz)L2_, respectively (Figure S10). In addition, from these values and using the ex situ HPLC technique, the subproducts of the reaction have been determined.

Acetaldehyde (CH_3_CHO) and acetic acid or acetate ion (C_2_H_3_O_2_^–^) are the main reaction compounds often obtained.^65^ Traces of CO_2_ (or carbonate CO_3_^–2^) are also often obtained. In this case, the main product of the reaction at the nanocatalysts is C_2_H_3_O_2_^–^ (inset in Figure S10). Considering only the reaction product detected by HPLC analysis, a general mechanism for the conversion of ethanol to C_2_H_3_O_2_^–^ on the nanocatalysts can be proposed according to the following reaction
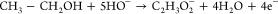
21

CH_3_CHO and CO_3_^–2^ have not been detected. In the case of the former, it has been reported that it reacts to produce C_2_H_3_O_2_^–^ through nucleophilic attack by OH^–^.^66^ For future work, the use of an in situ technique such as infrared spectroscopy is proposed to study the production of intermediates from the reaction.

In this work, the configuration chosen for the tests in the AEM-DEFC is that of the same nanocatalysts at the anode and cathode of each membrane-electrode assembly. It must be highlighted that the small-scale, homemade ethanol/O_2_ Teflon cell has been built to compare the performance of the nanocatalysts under the experimental conditions imposed. Therefore, it is difficult to make a comparison with other works from the literature unless a similar cell is reported.

**Figure 7:**
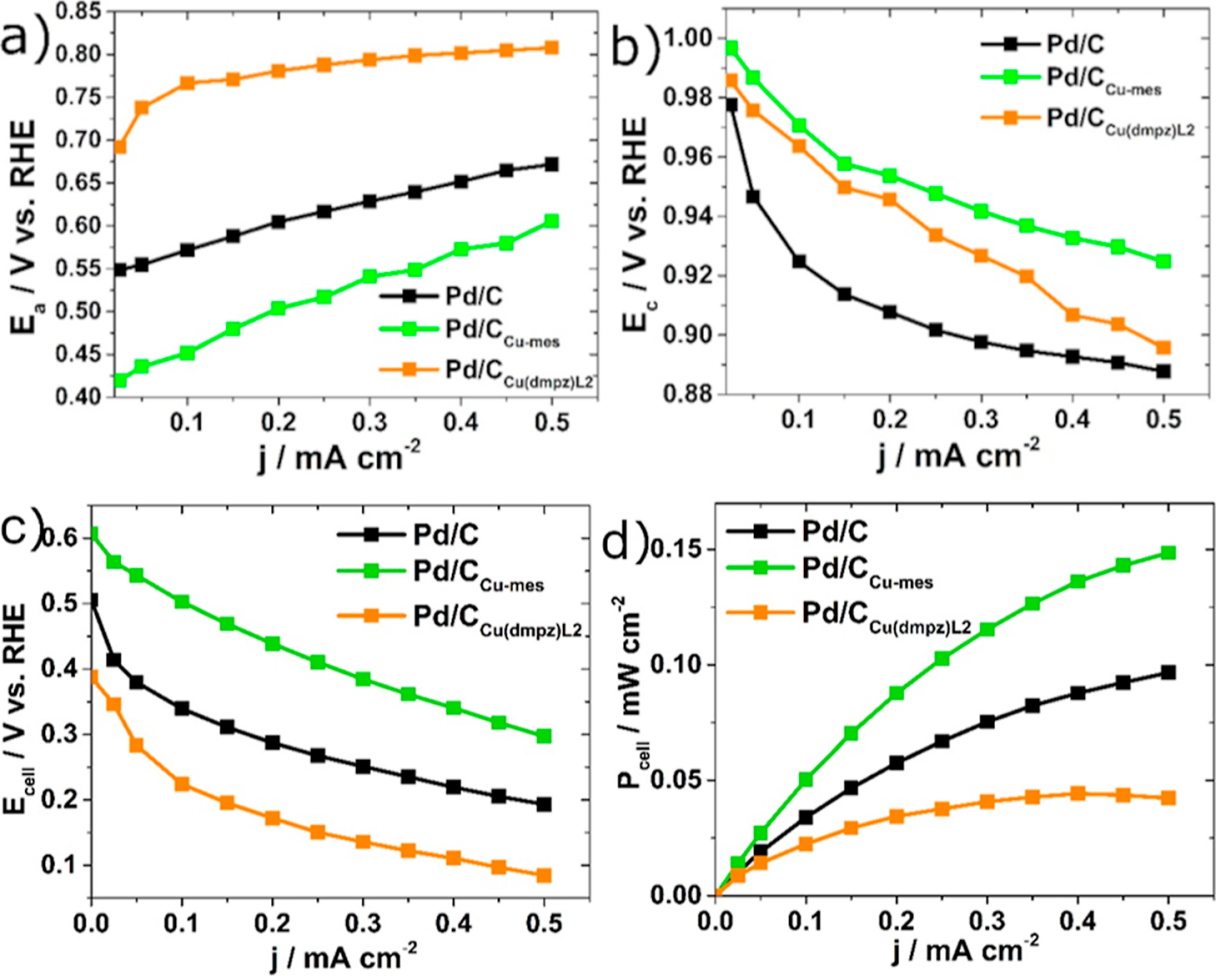
Individual polarization curves of the nanocatalysts showing the behavior oxf (a) the anode potential, *E*_a_, and (b) the cathode potential, *E*_c_. (c) Polarization curves and (d) power density curves of the AEM-DEFC equipped with the nanocatalysts as anodes and cathodes. Fuel: 0.5 mol L^–1^ EtOH + 0.5 mol L^–1^ KOH. AEM: Fumatech FAA. Feed at the cathode: O_2_ + 0.5 mol L^–1^ KOH.

Figure 7 shows the individual polarization curves of Pd/C, Pd/_CCu-mes_, and Pd/C_Cu(dmpz)L2_ showing the behavior of (a) the anode potential (*E*_a_) and (b) the cathode potential (*E*_c_) in fuel cell configuration (i.e., the plots show the potential difference between anode or cathode and the reference electrode, as each electrode is polarized over a given *j* interval).

Pd/C_Cu-mes_ shows an enhanced performance as anode, with an open circuit potential (OCP_a_) of 0.42 V/RHE, a value 0.13 and 0.26 V lower than Pd/C and Pd/C_Cu(dmpz)L2_, respectively (Table 1). Therefore, the overpotential of the EOR at Pd/C_Cu-mes_ is significantly smaller than that of the other nanocatalysts, while its high performance is sustained over the whole polarization curve. Opposite to that, Pd/C_Cu(dmpz)L2_ shows the poorest performance among the three nanocatalysts.

**Table 1:** Electrochemical Parameters of the AEM-DEFC Equipped with Pd/C, Pd/C_Cu-mes_, and Pd/C_Cu(dmpz)L2_ Anodes and Cathodes

nanocatalyst	OCP_a_ (V/RHE)	OCV (V)	*E*_cell_ at 0.25 mA cm^–2^ (V/RHE)	*P*_cell_ (mW cm^–2^)
Pd/C	0.55	0.50	0.27	0.09
Pd/C_Cu-mes_	0.42	0.60	0.43	0.14
Pd/C_Cu(dmpz)L2_	0.68	0.38	0.15	0.04

Pd/C_Cu-mes_ also show a better performance in the polarization curve as cathode, with an OCP_c_ of 1.00 V/RHE, followed by 0.99 and 0.98 V/RHE of Pd/C_Cu(dmpz)L2_ and Pd/C, respectively. This means that the overpotential of the ORR at Pd/C_Cu-mes_ over the scan is clearly lower compared to those of the other nanocatalysts. Interestingly, Pd/C_Cu-mes_ shows an improved behavior as a cathode than Pd/C, having a less significant potential drop as *j* increases.

The cell voltage (*E*_cell_) vs *j* polarization curves of the AEM-DAFC are shown in Figure 7c. Pd/CCu-mes as anode and cathode nanocatalyst generate the highest open circuit voltage (OCV), with a value of 0.60 V, followed by Pd/C and Pd/CCu(dmpz)L2, as shown in Table 1. It is to be highlighted that the *E*_cell_ values of the AEM-DEFC having Pd/CCu-mes are significantly higher compared to the other nanocatalysts at each *j* value. For example, the *E*_cell_ at 0.25 mA cm^–2^ is 0.43 with this nanocatalyst, compared to 0.27 and 0.15 V with Pd/C and Pd/CCu(dmpz)L2, respectively (Table 1). This outcome is a result of the high performance of Pd/CCu-mes as anode and cathode.

Meanwhile, Pd/C_Cu(dmpz)L2_ shows lower performance than Pd/C in the AEM-DEFC, a behavior ascribed mainly to its poor catalytic activity as an anode (Figure 7a). The poor performance of Pd/C_Cu(dmpz)L2_ in the AEM-DEFC differs from its catalytic performance in Figure 5c. Although more studies may be needed to explain this behavior, it is hypothesized that the Pd/C_Cu(dmpz)L2_ nanocatalyst/electrolyte interface failed to facilitate a fast transfer of species due to the EOR, probably because of the solid nature of the membrane. The low production of C_2_H_3_O_2_^–^ at this nanocatalysts, regardless of its high ethanol consumption (Figure S10), may be an indicator to infer that it produces several other reaction intermediates that poison its catalytic sites.

The maximum cell power density (*P*_cell_) from the AEM-DEFC is 0.14 mW cm^–2^ (Table 1) generated when Pd/C_Cu-mes_ is used as the anode and cathode. In view of the behavior of the AEM-DEFC, Pd/C_Cu-mes_ is a high-performance nanocatalyst with application as an anode and cathode in an alkaline environment.

## 4 Conclusions

The successful functionalization of Vulcan with Cu-mes and Cu(dmpz)L2 resulted in the formation of oxygenated functional groups and Cu sites on its surface, preserving sp^2^ nanodomains at Vulcan. XPS characterization revealed the formation of Cu^+^ and Cu^2+^ species, along with PdCu alloyed phases. This modified electronic structure of Pd, along with the bifunctional mechanism, enhanced the tolerance to CO poisoning and catalytic activity for the EOR of Pd/C_Cu-mes_ and Pd/C_Cu(dmpz)L2_ compared to Pd/C. Pd/C_Cu-mes_ exhibited improved electrochemical stability in home-built AEM-DEFC tests, showing higher ethanol consumption and acetate ion production, compared to Pd/C and Pd/C_Cu(dmpz)L2_. In a complete fuel cell analysis, Pd/C_Cu-mes_ generated the highest OCV and *P*_cell_ values, demonstrating that the functionalization of Vulcan with Cu-mes generates active surface sites that catalyze the EOR in the alkaline membrane environment. These findings highlight Pd/C_Cu-mes_ as a high-performance anode (and cathode) in the AEM-DEFC devices.

## Supporting Information Available

FTIR, Raman, XPS spectra, and parameters of functionalized carbon supports; characterization of the nanocatalysts by EDS, XRD, HRTEM, Raman, XPS; electrochemical evaluation: CO-stripping, catalytic activity for the EOR, AEM-DEFC performance in terms of ethanol consumption; and comparison of catalytic behavior (PDF)
