# Supplementary material for: Enhancing the Catalytic Activity of Pd Nanocatalysts for Anion Exchange Membrane Direct Ethanol Fuel Cells by Functionalizing Vulcan XC-72 with Cu Organometallic Compounds
Source: ACS Appl Nano Mater. 2024 Aug 20;7(17):20071–84. doi: 10.1021/acsanm.4c02670 (PMC11406490; doi:10.1021/acsanm.4c02670)
Supplement: Supplementary file 1 — an4c02670_si_001.pdf [file an4c02670_si_001.pdf]

Supporting Information

Enhancing the Catalytic Activity of Pd Nanocatalysts for Anion Exchange Membrane Direct Ethanol Fuel Cells by Functionalizing Vulcan XC-72 with Cu Organometallic Compounds

P.C. Meléndez-González[1], M.O. Fuentez-Torres[1], M.E. Sánchez-Castro[1,2], I.L. Alonso-Lemus[3], B. Escobar-Morales[4], W.J. Pech-Rodríguez[5], Teko W. Napporn[6], F.J. Rodríguez-Varela[1,2,*].

[1]Nanociencias y Nanotecnología, Cinvestav Unidad Saltillo, Av. Industria Metalúrgica 1062, Parque Industrial Ramos Arizpe. Ramos Arizpe, Coahuila, C.P 25900, México.
[2]Sustentabilidad de los Recursos Naturales y Energía, Cinvestav Unidad Saltillo.
[3]CONAHCYT-Cinvestav Saltillo. Sustentabilidad de los Recursos Naturales y Energía, Cinvestav Unidad Saltillo. Av. Industria Metalúrgica 1062, Parque Industrial Ramos Arizpe. Ramos Arizpe, Coahuila, C.P 25900, México.
[4]CONAHCyT, Centro de Investigación Científica de Yucatán, Unidad de Energía Renovable, Calle 43, No. 130 Col. Chuburná de Hidalgo, C. P. 97200, Mérida, Yucatán, México.
[5]Universidad Politécnica de Victoria, Av. Nuevas Tecnologías 5902, Parque Científico y Tecnológico de Tamaulipas, Cd Victoria, Tamaulipas, C.P. 87138, México
[6]Université de Poitiers, IC2MP UMR 7285 CNRS, « Equipe SAMCat», 4, rue Michel Brunet, B27, TSA 51106, 86073 Poitiers Cedex 09, France.
*E-mail: javier.varela@cinvestav.edu.mx

S1. FTIR, Raman, and XPS spectra of C, $C_{Cu\text{-}mes}$, and $C_{Cu(dmpz)L2}$.

Figure S1 shows the FTIR spectra of the supports. Non-functionalized C shows a band at around 3440 cm^{-1} due to the OH-stretching vibration from hydroxyl groups. It also displays the band at 2900 cm^{-1} attributed to C-H vibrations of alkane and alkyl groups. The band at 2368 cm^{-1} corresponds to the stretching of O-H bonds and vibrations of COOH groups. In the 1700–1200 cm^{-1} range, bands attributed to stretching vibrations of C-O species in carbonyl $HCOO^-$ as well as carboxyl groups are detected [1, 2]. These bands emerge due to the functional groups already developed at Vulcan.

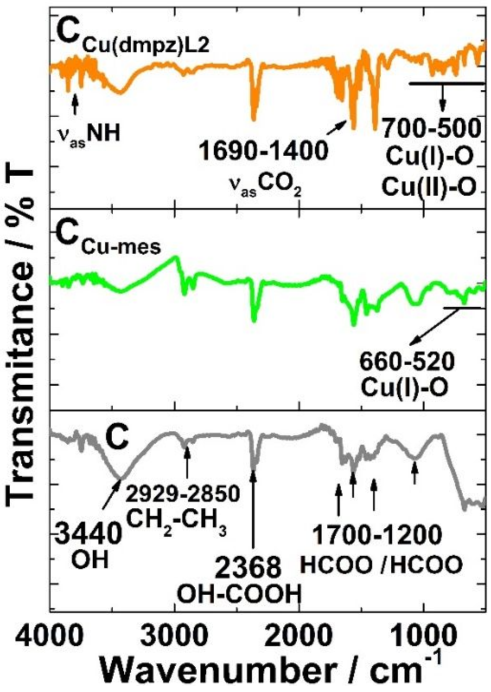

Figure S1. FT-IR spectra of C, $C_{Cu\text{-}mes}$ and $C_{Cu(dmpz)L2}$.

The FTIR spectrum of $C_{Cu\text{-}mes}$ shows a band assigned to Cu-O stretching vibrations in the 660-520 cm^{-1} range [3, 4]. Moreover, the spectrum of $C_{Cu(dmpz)L2}$ shows the characteristic signals ascribed to carboxylate and NH bands due to the (dmpz) ligand, which suggests the correct coordination of the compound [5].

The Raman spectra of C, $C_{Cu\text{-}mes}$, and $C_{Cu(dmpz)L2}$ are shown in Figure S2. The plots display the D and G bands characteristic of carbon materials at 1324 and 1584 cm^{-1}, respectively. In addition, the D' (~1615 cm^{-1}), D'' (~1500 cm^{-1}), and D* (~1170 cm^{-1}) interbands have been developed from the deconvolution of the spectra. The D band corresponds to sp^3 hybridization, directly related to impurities in the carbon structure. On the other hand, the G band is attributed to the sp^2 hybridization of carbon, characteristic of graphitic materials. The ratio of the intensity of the D and G bands (I_D/I_G) is proportional to the degree of sp^3/sp^2 hybridization of the carbon atoms [6, 7].

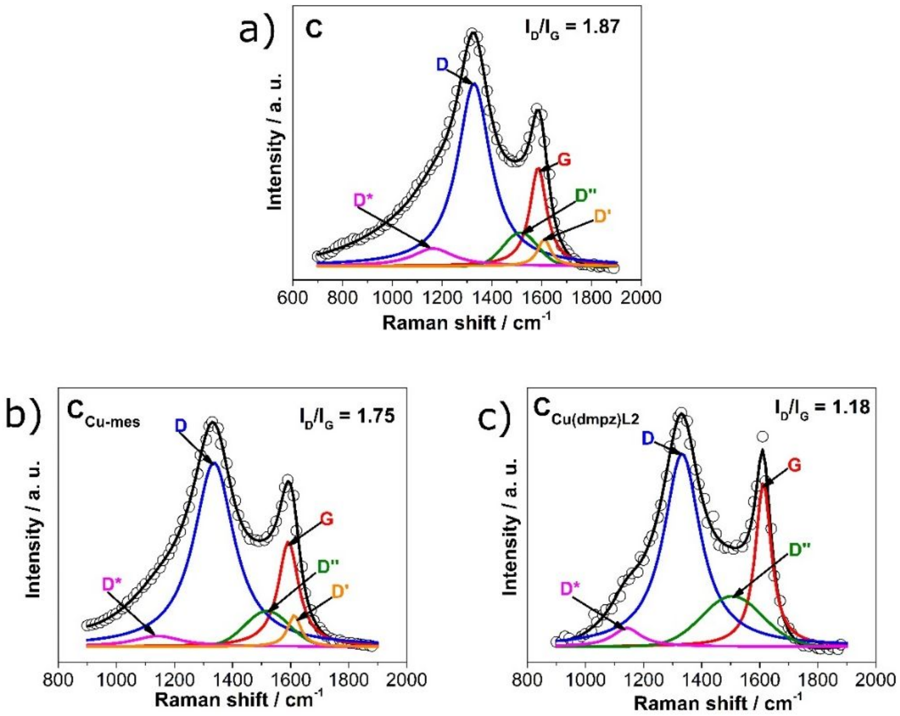

Figure S2. Raman spectra of a) C, b) $C_{Cu\text{-}mes}$ and c) $C_{Cu(dmpz)L2}$.

In Figure S2 a), C has an I_D/I_G ratio of 1.87, i.e., it is a carbon with relatively high structural disorder. Meanwhile, the intensity of the G band increases at $C_{Cu\text{-}mes}$ and $C_{Cu(dmpz)L2}$ compared to C (spectra in Figures S2 b and c, respectively), so their I_D/I_G ratio decreases to 1.75 and 1.18 (Table S1), respectively, due to an increase in sp^2 domains. Such changes at $C_{Cu\text{-}mes}$ and $C_{Cu(dmpz)L2}$ are attributed to an effect of π-π bonds between the graphitic structure of C and the Cu organometallic compounds, leading to constructive rehybridization that increases the sp^2 nanodomains at C during functionalization [8, 9].

The D’ interband usually merges with the G band and is due to the phonon confinement caused by structural defects [10]. In the case of $C_{Cu(dmpz)L2}$, D' is not detected due to overlapping with its G band (~1609 cm^{-1}), which is confirmed by the shift of the latter compared to C (G= ~1588 cm^{-1}), and $C_{Cu\text{-}mes}$ (G= ~1589 cm^{-1}). This is a particular modification of the carbon structure resulting from its functionalization with Cu(dmpz)L2, which differs from that with Cu-mes. Meanwhile, the D’’ interband has been assigned to interstitial defects of amorphous carbon fragments containing sp^2 bonds [11]. On this matter, the relative intensity of D’’ is 19, 22, and 26 for C, $C_{Cu\text{-}mes}$, and $C_{Cu(dmpz)L2}$,

respectively. Such an increase in D'' at the functionalized carbons is assigned to the presence of organic fragments and functional groups in their structure [12].

Moreover, the D* interband can be attributed to the stretching vibration of the sp^2-sp^3 (C-C and C=C) bonds [13], and the I_{D*}/I_G ratio has been determined as 0.17, 0.09, and 0.11 for C, $C_{Cu\text{-}mes}$, and $C_{CudmpzL2}$, respectively as seen in Table S1. Thus, it can be confirmed that the presence of crystalline phases increases after functionalization of Vulcan with the organometallic compounds.

Table S1. Raman data of C, $C_{Cu\text{-}mes}$, and $C_{CudmpzL2}$.

Nanocatalyst	Raman Shift / cm^{-1}					I_D/I_G	$I_{D''}$	I_{D*}/I_G
	D	G	D'	D''	D*			
C	1327	1588	1607	1519	1154	1.87	19	0.17
$C_{Cu\text{-}mes}$	1332	1589	1614	1519	1140	1.75	22	0.09
$C_{Cu(dmpz)L2}$	1325	1609	-	1497	1137	1.18	26	0.11

- Not determined.

Figure S3 displays the XPS spectra of $C_{Cu\text{-}mes}$. Its C 1s region is deconvoluted into three peaks (Figure S3 a) with the most intense signal being that of the sp^2 hybridization (C=C bond, BE= 284.71 eV) as seen in Table S2, followed by the less intense sp^3 hybridization (C-C bond, BE= 285.63 eV), and the C-O-C species (BE= 286.62 eV). Their relative concentration is 75.2, 17.6, and 7.1 at %, respectively (Table S2). The presence of sp^2 C=C bonds is due to graphitic phases in the structure of $C_{Cu\text{-}mes}$, which is in good agreement with the Raman spectra in Figure 2.

Figure S3 b) shows the O 1s region of $C_{Cu\text{-}mes}$, having signals at 530.69 and 531.73 eV assigned to CuO (9.0 at. %) and Cu_2O (27.7 at. %) bonds, respectively, confirming copper and oxygen interactions [14]. It is important to note that these signals do not appear in the O 1s region of non-functionalized Vulcan shown in [15]. It also shows signals ascribed to C=O (BE= 532.61 eV) and C-O (BE= 533.79 eV) bonds. The relative concentration of the former is higher compared to that of the latter (43.8 and 19.5 at. %, respectively, Table S2).

The deconvoluted Cu 2p region of $C_{Cu\text{-}mes}$ is shown in Figure S3 c). The signals centered at 935.02 and 954.81 eV are associated with the cuprous ion (Cu^{+}) in the Cu $2p^{3/2}$ and Cu $2p^{½}$ states, respectively. The peaks at 937.43 and 957.85 eV correspond to the Cu^{2+} species also in both states. The presence of satellite signals in the 940-950 eV and 960-970 eV regions indicates a significant contribution of Cu oxidized species in the spectra [16, 17]. These results agree with the identification of Cu oxides from XRD analysis (see Figure 1 a).

Figure S3 also shows the XPS spectra of $C_{Cu(dmpz)L2}$. The most intense signal in the C 1s region (Figure S3 d) is due to the sp^2 hybridization (C=C bond, BE=284.63 eV). Less intense signals correspond to the C-C, C-O-C, and C=O species (285.26, 286.39, and 287.50 eV, respectively, Table S2). The C=O peak is related to functional groups wherein oxygen is attached to the carbon by a double bond, such as quinine, carbonyl, carboxylic, and lactone groups [18]. It is important to note that this peak does not appear in the deconvoluted C 1s region of $C_{Cu\text{-}mes}$ and emerges most probably due to the concentration of carboxylates (5.7 at. %) which are found within the chemical structure of $C_{Cu(dmpz)L2}$. The deconvoluted peaks in Figure S3 have estimated FWHM (Table S2) close to what is reported in the literature [19-21].

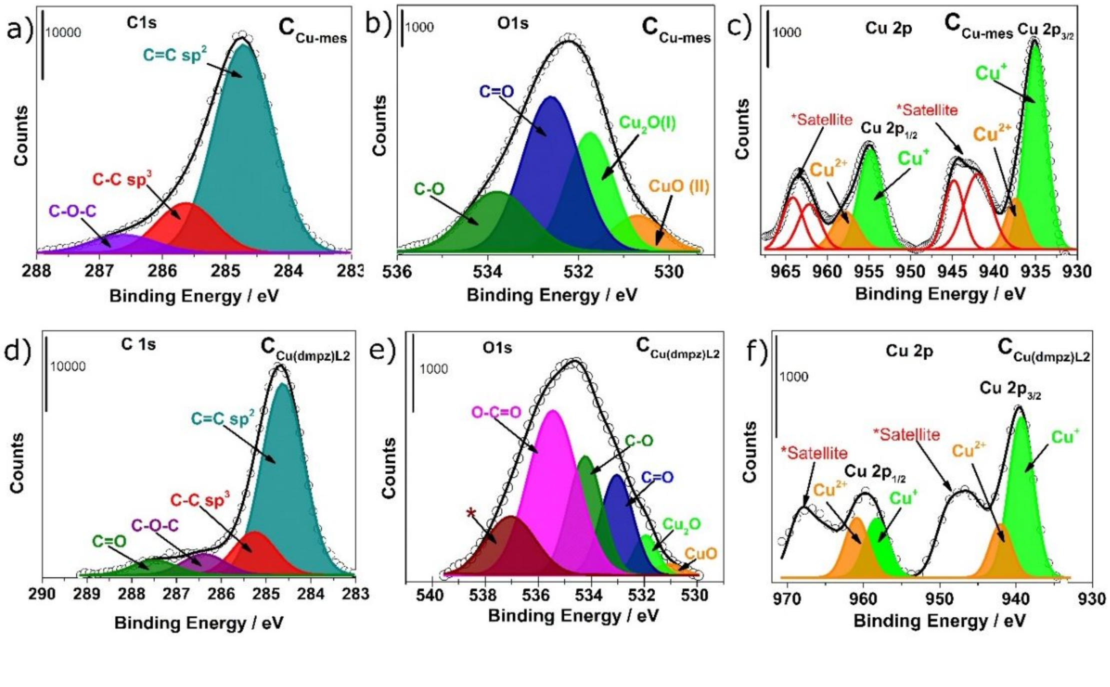

Figure S3. XPS spectra of $C_{Cu\text{-}mes}$ in the a) C 1s, b) O 1s and c) Cu 2p regions, and $C_{Cu(dmpz)L2}$ in the d) C 1s, e) O 1s and f) Cu 2p regions.

The O 1s region (Figure S3 e) at $C_{Cu(dmpz)L2}$ shows two low-intensity peaks assigned to CuO (BE=532.04 eV), and Cu_2O (BE= 531.94 eV), as in the case $C_{Cu\text{-}mes}$. The C=O, C-O, and O-C=O (not observed at $C_{Cu\text{-}mes}$) species are also detected at 533.04, 534.23, and 535.45 eV, respectively. The relative concentration of the species is shown in Table S2. The peak at 537.04 eV is attributed to traces of the (-O-C(O)-C_6H_4-C(O)-O- CH_2-CH_2-)$_n$ species from the organometallic compound, which has been reported in that BE elsewhere [22]. It is important to note the shift to higher BE of the C=O and C-O bonds of $C_{Cu(dmpz)L2}$ compared to $C_{Cu\text{-}mes}$ (Table S2), which are related to the specific interactions of the compounds with the carbon support. The identification of the O-C=O and (-O-C(O)-C_6H_4-C(O)-O- CH_2-CH_2-)$_n$ species can be correlated with the features of the XRD pattern of $C_{Cu(dmpz)L2}$ in Figure 3.

Figure S3 f) shows that the Cu 2p region of $C_{Cu(dmpz)L2}$ can be deconvoluted into the Cu^+ and Cu^{2+} species. The Cu^+ peaks are located at 938.67 and 958.73 eV, respectively, corresponding to the Cu $2p_{3/2}$ and Cu $2p_{1/2}$ states [23]. The peaks centered at 941.2 eV and 943 eV are those of Cu^{2+} in both Cu states. Satellites are also displayed at $C_{Cu(dmpz)L2}$.

Table S2. XPS parameters of C_{Cumes} and $C_{Cu(dmpz)L2}$.

**Support**	**State**	**Species**	**BE / eV**	**FWHM / eV**	**Composition / at. %**
$C_{Cu\text{-}mes}$	C 1s	C=C sp^2	284.71	1.10	75.2
	C 1s	C-C sp^3	285.63	1.08	17.6
	C 1s	C-O-C	286.62	1.20	7.1
	O 1s	CuO	530.69	1.16	9.0
	O 1s	Cu_2O	531.73	1.02	27.7
	O 1s	C=O	532.61	1.40	43.8
	O 1s	C-O	533.79	1.48	19.5
	Cu $2p_{3/2}$	Cu^+	935.02	3.60	45.5
	Cu $2p_{3/2}$	Cu^{2+}	937.43	3.05	7.0
	Cu $2p_{1/2}$	Cu^+	954.81	2.61	38.1
	Cu $2p_{1/2}$	Cu^{2+}	957.85	3.19	9.6

$C_{Cu(dmpz)L2}$	C 1s	C=C sp^2	284.63	1.10	70.7
	C 1s	C-C sp^3	285.26	1.10	15.8
	C 1s	C-O-C	286.39	1.12	7.8
	C 1s	C=O	287.50	1.15	5.7
	O 1s	CuO	531.04	1.18	1.5
	O 1s	Cu_2O	531.94	1.03	4.7
	O 1s	C=O	533.03	1.43	16.6
	O 1s	C-O	534.23	1.49	20.5
	O 1s	O-C=O	535.45	2.20	41.7
	O 1s	$(-O-C(O)-C_6H_4-C(O)-O-CH_2-CH_2-)_n$	537.04	2.22	14.9
	Cu $2p_{3/2}$	Cu^+	938.67	3.72	34.7
	Cu $2p_{3/2}$	Cu^{2+}	940.47	3.05	17.3
	Cu $2p_{1/2}$	Cu^+	958.73	3.73	26.5
	Cu $2p_{1/2}$	Cu^{2+}	961.32	3.30	22.4

Table S3 summarizes the chemical composition of the supports. Vulcan has 89.86 and 10.14 (wt. %) of C and O, respectively [24]. Whereas C_{Cu-mes} have a content of 80.51, 12.89, and 6.59 (wt. %) of C, Cu, and O, respectively. It should be mentioned that the Cu content is lower than the theoretically expected value (i.e., 20 wt. %), and this may be due to the presence of copper complexes. Meanwhile, $C_{Cu(dmpz)L2}$ shows 71.31, 22.36, and 6.32 (wt. %) of C, Cu, and O, respectively. In this case, the concentration of Cu is only slightly lower than theoretically expected (25 wt. %).

Table S3. Chemical composition of C, $C_{Cu\text{-}mes}$, and $C_{Cu(dmpz)L2}$.

**Support**	C	O	Cu
	/ wt. %		
C	89.86	10.14	-
$C_{Cu\text{-}mes}$	80.51 ± 0.86	6.59 ± 0.60	12.89 ± 1.38
$C_{Cu(dmpz)L2}$	71.31 ± 0.18	6.32 ± 0.29	22.36 ± 0.37

– Not determined.

S2. Physicochemical and electrochemical properties of the nanocatalysts.

Table S4 shows the chemical composition of Pd/C, Pd/$C_{Cu\text{-}mes}$, and Pd/$C_{Cu(dmpz)L2}$ obtained from EDS analysis. Overall, Pd/C shows a chemical composition close to the theoretically expected, with a Pd content of 16.04 ± 0.93 (wt. %). As for Pd/$C_{Cu\text{-}mes}$, it has C and O concentrations of 69 ± 1.4 and 1.97 ± 0.2, the latter due to the formation of metal oxide or oxygen species on the carbon surface. Its Pd content is 19.18 ± 0.93, very close to the nominally expected value (20 wt. %) while the concentration of Cu from the functionalization is 9.34 ± 0.7 (wt. %). Even though this value is slightly lower when compared to $C_{Cu\text{-}mes}$ (Table S3), it confirms that Cu from the organometallic compound is stable even after being submitted to the reduction atmosphere of the polyol method during the synthesis of Pd/$C_{Cu\text{-}mes}$. In the case of Pd/$C_{Cu(dmpz)L2}$, Pd and Cu contents of 16.27 and 11.95 wt. %, respectively, have been determined. The Cu concentration decreases compared to $C_{Cu(dmpz)L2}$ in Table S3, i.e., a lower stability compared to Pd/$C_{Cu\text{-}mes}$. Meanwhile, a significantly higher O concentration is observed at Pd/$C_{Cu(dmpz)L2}$.

Table S4. Chemical composition of Pd, Pd/$C_{Cu\text{-}mes}$, and Pd/$C_{Cu(dmpz)L2}$.

**Nanocatalyst**	C	Pd	O	Cu
		/ wt. %		
Pd/C	78.3 ± 1.4	16.04 ± 0.93	5.6 ± 0.21	-
Pd/$C_{Cu\text{-}mes}$	69.42 ± 1.4	19.18 ± 0.93	1.97 ± 0.21	9.34 ± 0.71
Pd/$C_{Cu(dmpz)L2}$	52.35 ± 1.4	16.27 ± 1.01	19.45 ± 3.04	11.95± 1.08

- Not determined.

Table S5 shows structural (from the (111) Pd plane) and d (from the (220) Pd plane) values of the nanocatalysts.

Table S5. Structural parameters and d values of the nanocatalysts.

**Nanocatalyst**	(111) plane position / °	a_{fcc} / nm	D / %	d_{XRD} / nm	d_{TEM} / nm
Pd/C	40.30	0.387	-	7.9	11.44 ± 2.6
Pd/$C_{Cu\text{-}mes}$	41.57	0.375	31	3.4	6.12 ± 1.7
Pd/$C_{Cu(dmpz)L2}$	42.35	0.369	33	6.3	5.72 ± 1.52

Structural parameters and d_{XRD} were obtained from (111) and the (220) Pd planes, respectively.

- Not determined.

Figures S4-S6 show the High Angle Annular Dark Field (HAADF) images and chemical mapping of Pd/C, Pd/$C_{Cu\text{-}mes}$, and Pd/$C_{Cu(dmpz)L2}$, respectively. The nanocatalysts show the homogeneous dispersion of Pd nanoparticles. Moreover, the two latter have a large number of Pd and Cu nanoparticles overlapped at the same spots, supporting the findings by XRD of the formation of Pd-Cu alloyed phases. C and O are well dispersed as well at the nanocatalysts.

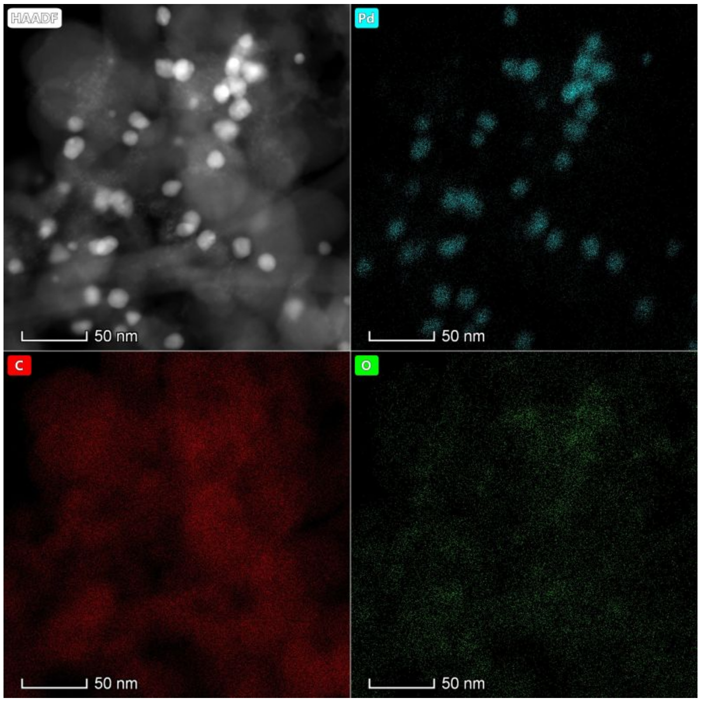

Figure S4. HAADF micrograph and elemental mapping of Pd/C.

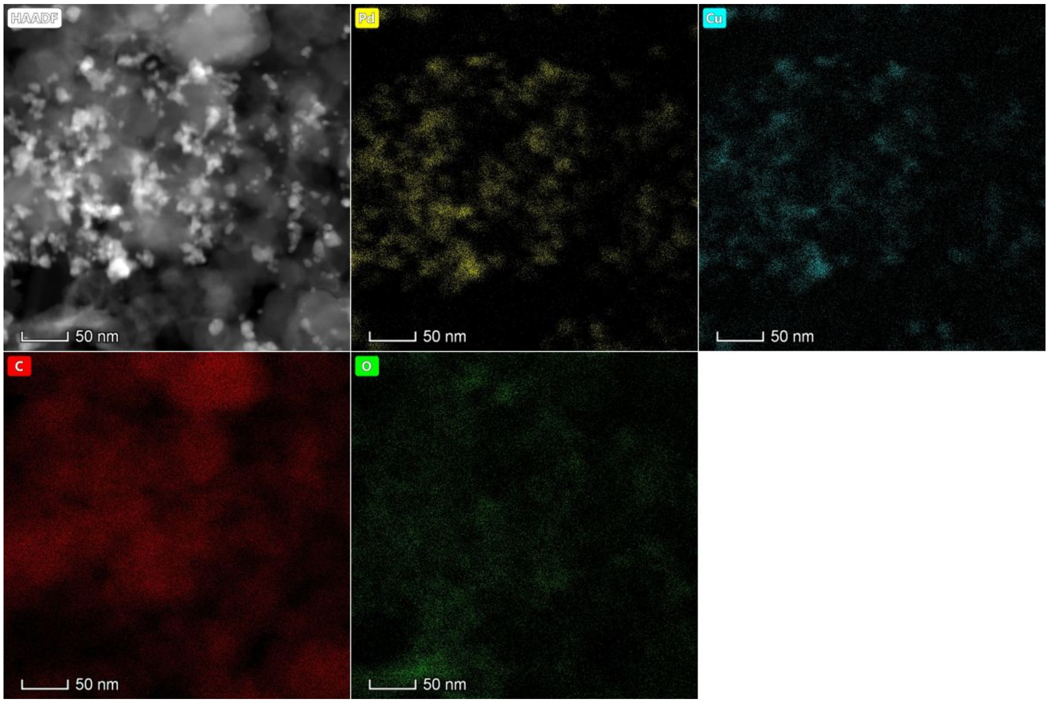

Figure S5. HAADF micrograph and elemental mapping of Pd/$C_{Cu\text{-}mes}$.

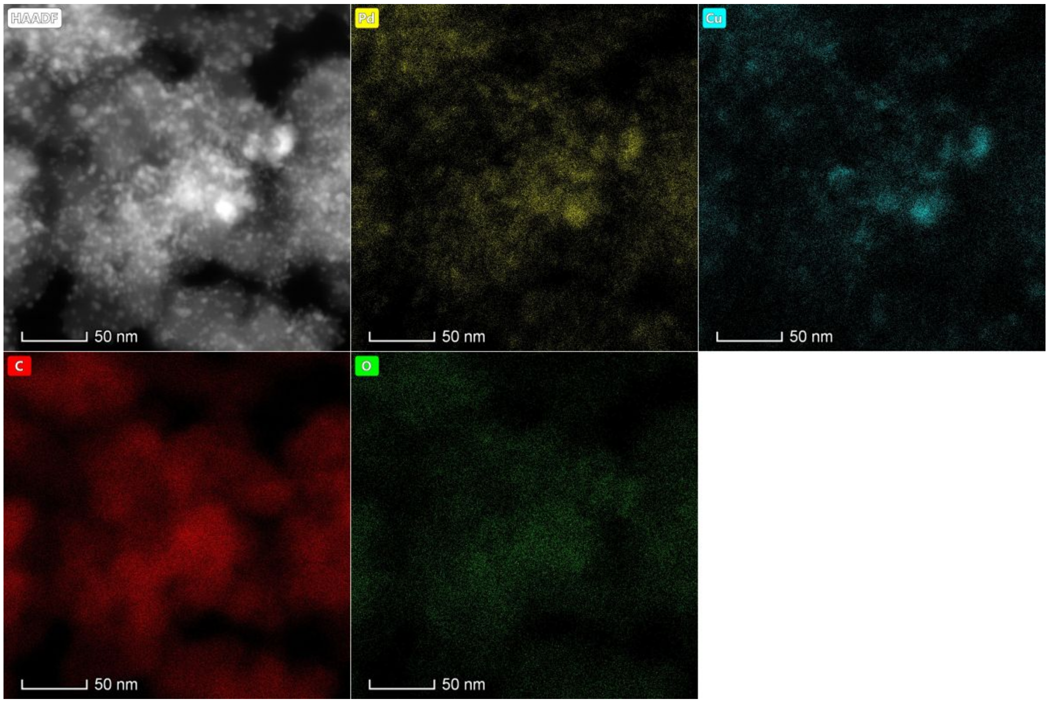

Figure S6. a) HAADF micrograph and elemental mapping of Pd/$C_{Cu(dmpz)L2}$.

The deconvoluted Raman spectra of Pd/C, Pd/C_{Cumes}, and Pd/$C_{Cu(dmpz)L2}$ are shown in Figures S7 a-c). The characteristic D and G bands already described in Figure S2 can be observed, as well as the D', D'', and D* interbands. The I_D/I_G ratio of Pd/C is 1.69 (Table S6), lower than C without Pd nanoparticles (1.87, Figure S2 a) suggesting an effect of the dispersion of Pd nanoparticles on the sp^2 domains of the support during the synthesis of the nanocatalyst. Such an effect has been discussed previously [25].

The I_D/I_G ratio of Pd/$C_{Cu\text{-}mes}$ increases to 1.92 from 1.75 at $C_{Cu\text{-}mes}$ (Tables S6 and S2, respectively). This same effect is observed when comparing Pd/$C_{Cu(dmpz)L2}$ and $C_{Cu(dmpz)L2}$, i.e., the ratio increases to 1.80 related to 1.18 for the latter. Some authors have attributed the increase in the I_D/I_G ratio of the carbon support with the addition of Pd nanoparticles to two possible reasons: i) the removal of oxygenated groups generates a re-establishment of the graphitic lattice, producing a higher concentration of sp^3 nanodomains; and ii) the nanoparticles can be treated as defects that induce structural disorder, which is undesirable but plays an important role in the development of catalytic activity [26-29].

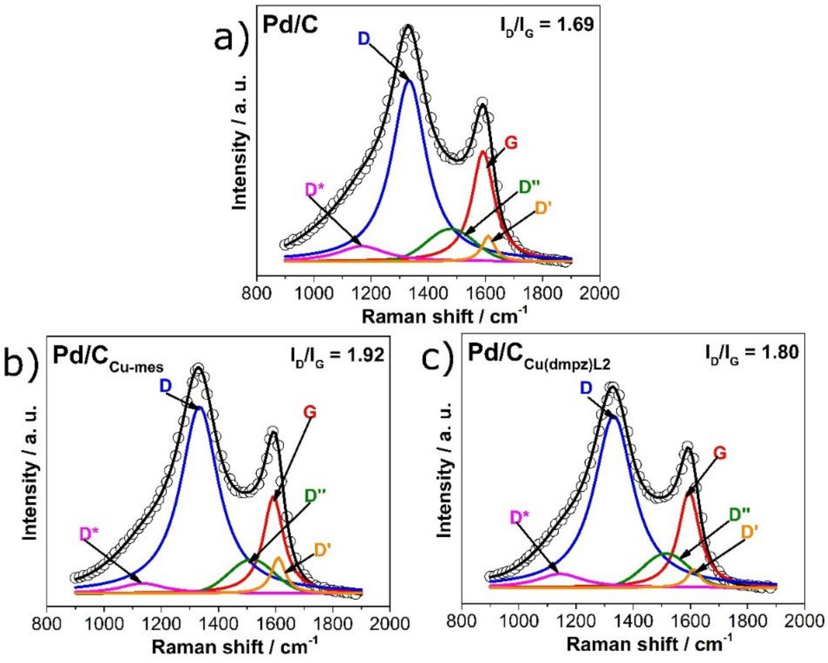

Figure S7. Raman spectra of a) Pd/C, b) Pd/$C_{Cu\text{-}mes}$, and c) Pd/$C_{Cu(dmpz)L2}$.

The relative intensity of D'' is 22.1, 27.2, and 28.4 for Pd/C, Pd/$C_{Cu\text{-}mes}$, and Pd/$C_{Cu(dmpz)L2}$ respectively. As can be seen in Table S6, it is higher at Pd/$C_{Cu\text{-}mes}$ and Pd/$C_{Cu(dmpz)L2}$ which suggests a modification on sp^3 domains during the synthesis of the nanocatalysts due to the presence of Pd nanoparticles.

Table S6. Raman data of Pd/C, Pd/$C_{Cu\text{-}mes}$ and Pd/$C_{Cu(dmpz)L2}$.

**Nanocatalyst**	**Raman Shift / cm^{-1}**					**I_D/I_G**	**$I_{D''}$**	**I_{D*}/I_G**
	D	G	D'	D''	D*			
Pd/C	1330	1592	1613	1485	1163	1.69	22.1	0.15
Pd/$C_{Cu\text{-}mes}$	1332	1597	1611	1516	1130	1.92	27.2	0.11
Pd/$C_{Cu(dmpz)L2}$	1332	1597	1611	1508	1135	1.80	28.4	0.13

Moreover, the I_{D*}/I_G ratio has been determined as 0.15, 0.11, and 0.13 for Pd/C, Pd/$C_{Cu\text{-}mes}$ and Pd/$C_{Cu(dmpz)L2}$, respectively (Table S6). In this case, it decreases for nanocatalysts supported on

functionalized carbon confirming the modification of the sp^3 domains. This may be related to an increase in disorder and surface area. Such modification can in turn lead to an increase in catalytic activity [30].

Table S7. XPS parameters of $Pd/C_{Cu\text{-}mes}$ and $Pd/C_{Cu(dmpz)L2}$.

Nanocatalyst	State	Species	BE / eV	FWHM / eV	Composition / at. %
**$Pd/C_{Cu\text{-}mes}$**	Pd $3d_{5/2}$	PdCu	335.00	0.76	13.6
	Pd $3d_{5/2}$	Pd^0	335.54	0.67	23.3
	Pd $3d_{5/2}$	Pd^{2+}	336.63	0.60	12.8
	Pd $3d_{5/2}$	Pd^{4+}	338.58	1.00	13.6
	Pd $3d_{3/2}$	PdCu	340.26	0.76	6.1
	Pd $3d_{3/2}$	Pd^0	340.71	0.66	12.0
	Pd $3d_{3/2}$	Pd^{2+}	341.94	0.57	7.7
	Pd $3d_{3/2}$	Pd^{4+}	341.62	1.00	11.6
	C 1s	C=C sp^2	284.77	1.06	77.5
	C 1s	C-C sp^3	285.73	1.07	16.6
	C 1s	C-O-C	286.77	1.10	5.9
	O 1s	CuO	529.94	1.30	2.4
	O 1s	Cu_2O	530.82	1.20	6.8
	O 1s	PdO	531.73	1.30	22.7
	O 1s	Pd_2O	532.52	1.22	19.6
	O 1s	C=O	533.30	1.01	29.5
	O 1s	C-O	534.23	1.10	12.0
	O 1s	O-C=O	535.10	1.28	6.7
	Cu $2p_{3/2}$	PdCu	932.13	1.28	12.1
	Cu $2p_{3/2}$	Cu^+	932.35	1.66	40.9
	Cu $2p_{3/2}$	Cu^{2+}	934.45	1.97	13.5
	Cu $2p_{1/2}$	PdCu	951.41	1.68	9.7
	Cu $2p_{1/2}$	Cu^+	952.33	1.55	14.0
	Cu $2p_{1/2}$	Cu^{2+}	954.22	2.0	9.8
**$Pd/C_{Cu(dmpz)L2}$**	Pd $3d_{5/2}$	PdCu	335.23	0.86	7.6
	Pd $3d_{5/2}$	Pd^0	335.74	0.81	28.1
	Pd $3d_{5/2}$	Pd^{2+}	336.42	1.10	19.4
	Pd $3d_{5/2}$	Pd^{4+}	338.24	1.57	9.3
	Pd $3d_{3/2}$	PdCu	340.70	0.84	6.5
	Pd $3d_{3/2}$	Pd^0	341.08	0.80	12.2
	Pd $3d_{3/2}$	Pd^{2+}	341.96	1.00	11.8

Pd $3d_{3/2}$	Pd^{4+}	342.52	1.33	5.1
C 1s	C=C sp^2	284.71	1.12	47.0
C 1s	C-C sp^3	285.49	1.33	21.1
C 1s	C-O-C	286.57	1.77	18.2
C 1s	C=O	287.76	1.49	7.5
C 1s	O-C=O	289.50	1.80	6.3
O 1s	CuO	530.15	1.70	4.1
O 1s	Cu_2O	531.12	1.45	7.4
O 1s	PdO	531.96	1.29	16.0
O 1s	Pd_2O	532.77	1.50	25.5
O 1s	C=O	533.59	1.28	20.6
O 1s	C-O	534.45	1.26	17.8
O 1s	O-C=O	535.64	1.74	8.6
Cu $2p_{3/2}$	PdCu	932.28	1.83	10.6
Cu $2p_{3/2}$	Cu^+	932.67	1.88	51.1
Cu $2p_{3/2}$	Cu^{2+}	935.06	2.00	7.1
Cu $2p_{1/2}$	PdCu	951.37	1.82	4.9
Cu $2p_{1/2}$	Cu^+	952.53	1.90	21.5
Cu $2p_{1/2}$	Cu^{2+}	954.71	2.00	4.8

Table S8. Electrochemical parameters of the CO-stripping and the EOR at the nanocatalysts.

**Nanocatalyst**	**$E_{onset,CO}$**	**E_{ox}**	**$ECSA_{CO}$**	**E_{onset}**	**j_m**	**$ECSA_{PdO}$**
	V/RHE		(m^2 g^{-1})	(V/RHE)	(mA mg^{-1}_{Pd})	(m^2 g^{-1}_{Pd})
**Before ADT**						
Pd/C	0.70	0.82	46.0	0.41	808.3	29.4
Pd/$C_{Cu\text{-}mes}$	0.19	0.79	102.8	0.38	1231.3	118.7
Pd/$C_{Cu(dmpz)L2}$	0.13	0.81	145.2	0.38	1001.8	148.0
**After ADT**						
Pd/C	-	-	-	0.41	925.0	15.0
Pd/$C_{Cu\text{-}mes}$	-	-	-	0.38	1078.0	63.9
Pd/$C_{Cu(dmpz)L2}$	-	-	-	0.38	1064.0	55.4

-Not determined

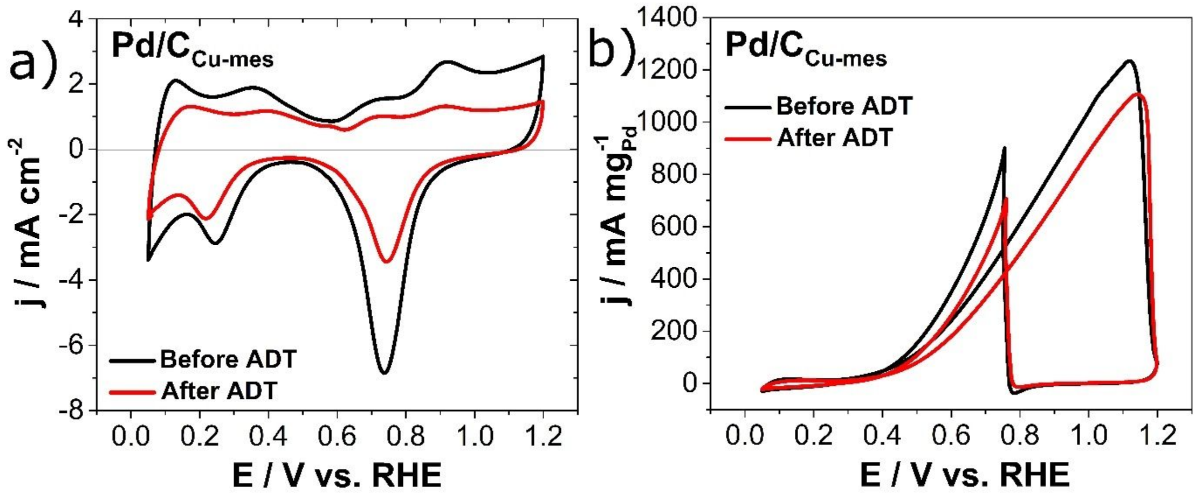

Figure S8. a) CVs of Pd/C$_{Cu-mes}$ in N_2-saturated 0.5 mol L^{-1} KOH recorded at 20 mV s^{-1} and b) polarization curves of Pd/C$_{Cu-mes}$ in 0.5 mol L^{-1} KOH + 0.5 mol L^{-1} EtOH recorded at 20 mV s before and after of ADT.

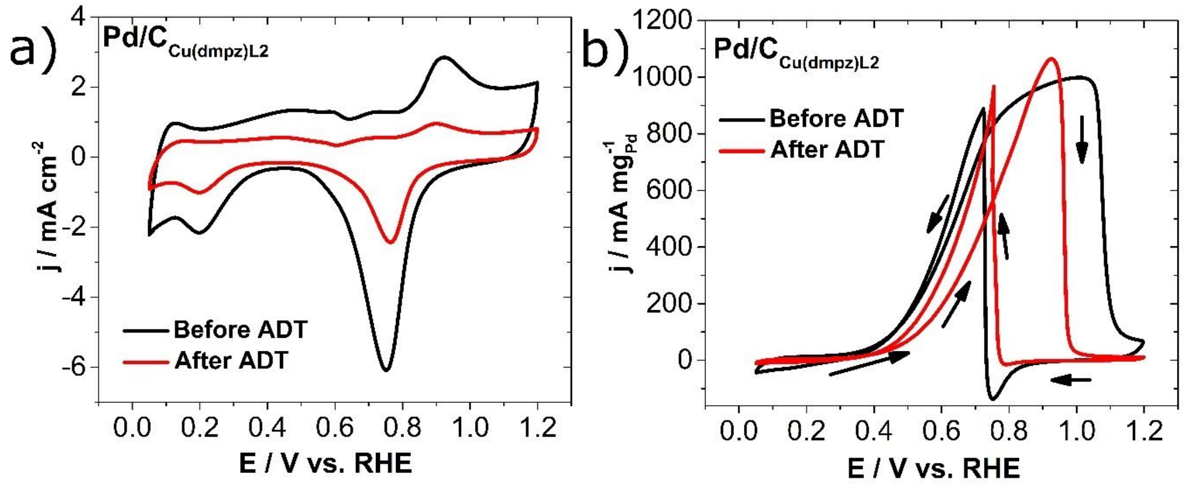

Figure S9. a) CVs of Pd/C$_{Cu(dmpz)L2}$ in N_2-saturated 0.5 mol L^{-1} KOH recorded at 20 mV s^{-1} and b) polarization curves of Pd/C$_{Cu(dmpz)L2}$ in 0.5 mol L^{-1} KOH + 0.5 mol L^{-1} EtOH recorded at 20 mV s^{-1} before and after of ADT.

Table S9. Electrochemical parameters of the EOR at Pd-based nanocatalysts.

Nanocatalyst	$ECSA_{PdO}$ ($m^2\ g^{-1}$)	E_{onset} (V/RHE)	j ($mA\ cm^{-2}$)	Electrolyte	Ref.
PdCu/C-DA15	97.5	0.36	108.6	1 mol L^{-1} NaOH + 1 mol L^{-1} EtOH	31
Pd-Cu/C	43.45	0.46	111.2	1 mol L^{-1} KOH + 1mol L^{-1} EtOH	32
Pd-NiO/MWCNT/rGO	42.05	0.44	90.8	1 mol L^{-1} KOH + 1mol L^{-1} EtOH	33
Pd/Ni/MOFDC	25.77	0.39	30.4	0.5 mol L^{-1} KOH + 0.5 mol L^{-1}EtOH	34
$PdAu_3/C$	-	0.56	54.5	1 mol L^{-1} KOH + 1 mol L^{-1} EtOH	35
Pt/PdCu	-	0.46	183.0	1 mol L^{-1} KOH + 1 mol L^{-1} EtOH	36
$Pd/C_{Cu\text{-}mes}$	118.7	0.38	119.6	0.5 mol L^{-1} KOH + 0.5 mol L^{-1}EtOH	This work
$Pd/C_{Cu(dmpz)L2}$	148.0	0.38	81.2		

-Not reported

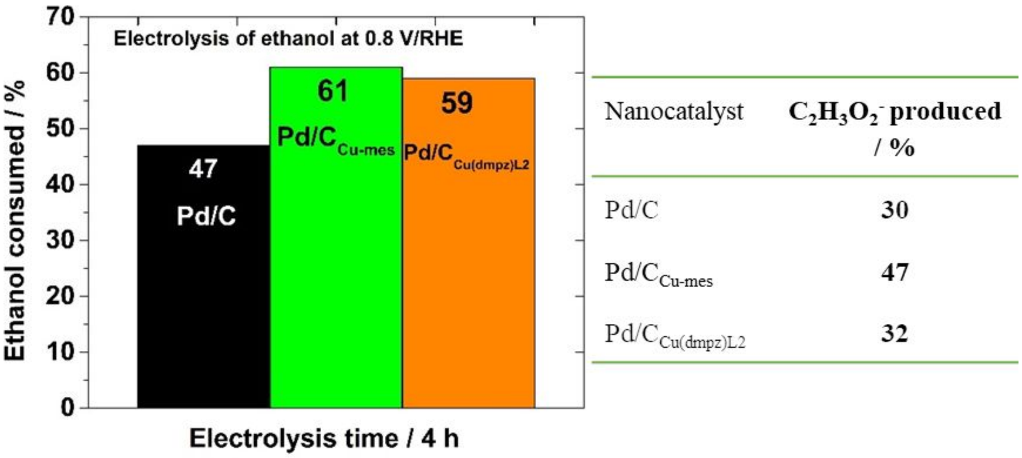

Figure S10. Ethanol consumed during 4 h at the nanocatalysts. Polarization potential: 0.8 V/RHE. Inset: $C_2H_3O_2^-$ produced from the reaction.

References

(1) Abidat, I.; Morais, C.; Pronier, S.; Guignard, N.; Comparot, J. D.; Canaff, C.; Napporn, T. W.; Habrioux, A.; Mamede, A. S.; Lamonier, J. F.; Kokoh, K. B. Effect of Gradual Reduction of Graphene Oxide on the CO Tolerance of Supported Platinum Nanoparticles. Carbon **2017**, 111, 849-858. DOI: 10.1016/j.carbon.2016.10.050

(2) Yin, S.; Shen, P. K.; Song, S.; Jiang, S. P. Functionalization of Carbon Nanotubes by an Effective Intermittent Microwave Heating-Assisted HF/H_2O_2 Treatment for Electrocatalyst Support of Fuel Cells. Electrochim. Acta **2009**, 54, 6954-6958. DOI: 10.1016/j.electacta.2009.07.009

(3) Ethiraj, A. S.; Kang, D. J. Synthesis and Characterization of CuO Nanowires by a Simple Wet Chemical Method. Nanoscale Res. Lett. **2012**, 7, 70. DOI: 10.1186/1556-276X-7-70

(4) Thekkae Padil, V. V.; Černík, M. Green Synthesis of Copper Oxide Nanoparticles using Gum Karaya as a Biotemplate and their Antibacterial Application. Int. J. Nanomedicine **2013**, 8, 889-898. DOI: 10.2147/IJN.S40599

(5) Sun, Y.-j.; Cheng, P.; Yan, S.-p.; Jiang, Z.-h.; Liao, D.-z.; Shen, P.-w. Synthesis, Crystal Structure and Properties of Novel Zinc(II) and Cobalt(II) Chain Complexes with 3,5-dimethylpyrazole and Thiocyanate. J. Coord. Chem. **2002**, 55, 363-372. DOI: 10.1080/714917024

(6) Alonso-Lemus, I. L.; Figueroa-Torres, M. Z.; Lardizabal-Gutíerrez, D.; Bartolo-Pérez, P.; Carrillo-Rodríguez, J. C.; Rodríguez-Varela, F. J. Converting Chicken Manure into Highly Active N–P Co-doped Metal-free Biocarbon Electrocatalysts: Effect of Chemical Treatment on their Catalytic Activity for the ORR. Sustain. Energy Fuels **2019**, 3, 1307-1316. DOI: 10.1039/C8SE00583D

(7) Martínez-Loyola, J. C.; Alonso-Lemus, I. L.; Sánchez-Castro, M. E.; Escobar-Morales, B.; Torres-Lubián, J. R.; Rodríguez-Varela, F. J. Surface Functionalization of Ordered Mesoporous Hollow Carbon Spheres with Ru Organometallic Compounds as Supports of Low-Pt Content Nanocatalysts for Alkaline Hydrogen and Oxygen Evolution Reactions. MRS Advances **2020**, 5, 2973-2989. DOI:10.1557/adv.2020.367.

(8) Siller-Ceniceros, A. A.; Sánchez-Castro, E.; Morales-Acosta, D.; Torres-Lubián, J. R.; Martínez-Guerra, E.; Rodríguez-Varela, J. Functionalizing Reduced Graphene Oxide with Ru-Organometallic Compounds as an Effective Strategy to Produce High-Performance Pt Nanocatalysts for the Methanol Oxidation Reaction. ChemElectroChem **2019**, 6, 4902-4916. DOI: 10.1002/celc.201901190

(9) Martínez-Loyola, J. C.; Siller-Ceniceros, A. A.; Sánchez-Castro, M. E.; Sánchez, M.; Torres-Lubián, J. R.; Escobar-Morales, B.; Ornelas, C.; Alonso-Lemus, I. L.; Rodríguez-Varela, F. J. High Performance Pt Nanocatalysts for the Oxidation of Methanol and Ethanol in Acid Media by Effect of Functionalizing Carbon Supports with Ru Organometallic Compounds. J. Electrochem. Soc. **2020**, 167, 164502. DOI 10.1149/1945-7111/abcabb

(10) Carrillo-Rodríguez, J. C.; Garay-Tapia, A. M.; Escobar-Morales, B.; Escorcia-García, J.; Ochoa-Lara, M. T.; Rodríguez-Varela, F. J.; Alonso-Lemus, I. L. Insight into the Performance and Stability of N-doped Ordered Mesoporous Carbon Hollow Spheres for the ORR: Influence of the Nitrogen Species on their Catalytic Activity after ADT. Int. J. Hydrogen Energy **2021**, 26087-26100. DOI: 10.1016/j.ijhydene.2021.01.047

(11) Claramunt, S.; Varea, A.; López-Díaz, D.; Velázquez, M. M.; Cornet, A.; Cirera, A. The Importance of Interbands on the Interpretation of the Raman Spectrum of Graphene Oxide. J. Phys. Chem. C **2015**, 119, 10123-10129. DOI: 10.1021/acs.jpcc.5b01590

(12) Goodman, P. A.; Li, H.; Gao, Y.; Lu, Y. F.; Stenger-Smith, J. D.; Redepenning, J. Preparation and Characterization of High Surface Area, High Porosity Carbon Monoliths from Pyrolyzed Bovine Bone and their Performance as Supercapacitor Electrodes. Carbon **2013**, 55, 291-298. DOI: 10.1016/j.carbon.2012.12.066

(13) Rebelo, S. L. H.; Guedes, A.; Szefczyk, M. E.; Pereira, A. M.; Araújo, J. P.; Freire, C. Progress in the Raman Spectra Analysis of Covalently Functionalized Multiwalled Carbon Nanotubes: Unraveling Disorder in Graphitic Materials. Phys. Chem. Chem. Phys. **2016**, 18, 12784-12796. DOI: 10.1039/C5CP06519D

(14) Wang, Y.; Lü, Y.; Zhan, W.; Xie, Z.; Kuang, Q.; Zheng, L. Synthesis of porous Cu_2O/CuO Cages using Cu-based Metal–organic Frameworks as Templates and their Gas-sensing Properties. J. Mater. Chem. A **2015**, 3, 12796-12803. DOI: 10.1039/C5TA01108F

(15) Moraes, A.; Assumpção, M. H. M. T.; Simões, F. C.; Antonin, V. S.; Lanza, M. R. V.; Hammer, P.; Santos, M. C. Surface and Catalytical effects on Treated Carbon Materials for Hydrogen Peroxide Electrogeneration. Electrocatalysis **2016**, 7, 60-69. DOI: 10.1007/s12678-015-0279-5

(16) Gao, D.; Zhang, J.; Zhu, J.; Qi, J.; Zhang, Z.; Sui, W.; Shi, H.; Xue, D. Vacancy-Mediated Magnetism in Pure Copper Oxide Nanoparticles. Nanoscale Res. Lett. **2010**, 5, 769. DOI: 10.1007/s11671-010-9555-8

(17) Maya-Cornejo, J.; Carrera-Cerritos, R.; Sebastián, D.; Ledesma-García, J.; Arriaga, L. G.; Aricò, A. S.; Baglio, V. PtCu Catalyst for the Electro-Oxidation of Ethanol in an Alkaline Direct Alcohol Fuel Cell. Int. J. Hydrogen Energy **2017**, 42, 27919-27918. DOI: 10.1016/j.ijhydene.2017.07.226

(18) Senthil Kumar, S. M.; Soler Herrero, J.; Irusta, S.; Scott, K. The Effect of Pretreatment of Vulcan XC-72R Carbon on Morphology and Electrochemical Oxygen Reduction Kinetics of Supported Pd Nano-Particle in Acidic Electrolyte. J. Electroanal. Chem. **2010**, 647, 211-221. DOI: 10.1016/j.jelechem.2010.05.021

(19) Batista, J.; Pintar, A.; Mandrino, D.; Jenko, M.; Martin, V. XPS and TPR Examinations of γ-Alumina-Supported Pd-Cu Catalysts. Appl. Catal. A: General **2001**, 206, 113-124. DOI: 10.1016/S0926-860X(00)00589-5

(20) Piedade, A. P.; Cangueiro, L. Influence of Carbyne Content on the Mechanical Performance of Nanothick Amorphous Carbon Coatings. Nanomaterials **2020**, 10, 780. DOI: 10.3390/nano10040780

(21) Mukhopadhyay, A. K.; Roy, A.; Bhattacharjee, G.; Das, S. C.; Majumdar, A.; Wulff, H.; Hippler, R. Surface Stoichiometry and Depth Profile of Ti_x-Cu_yN_z Thin Films Deposited by Magnetron Sputtering. Materials **2021**, 14, 3191. DOI: doi.org/10.3390/ma14123191

(22) Salem, J. R.; Sequeda, F. O.; Duran, J.; Lee, W. Y.; Yang, R. M. Solventless Polyimide Films by Vapor Deposition. J. Vac. Sci. Technol. A **1986** 4, 369–374. DOI: 10.1116/1.573930

(23) Jiang, P.; Prendergast, D.; Borondics, F.; Porsgaard, S.; Giovanetti, L.; Pach, E.; Newberg, J.; Bluhm, H.; Besenbacher, F.; Salmeron, M. Experimental and Theoretical Investigation of the Electronic Structure of Cu_2O and CuO Thin Films on Cu(110) using X-ray Photoelectron and Absorption Spectroscopy. J. Chem. Phys. **2013**, 138, 024704. DOI: https://doi.org/10.1063/1.4773583

(24) García-Mayagoitia, S.; Fernández-Luqueño, F.; Morales-Acosta, D.; Carrillo-Rodríguez, J. C.; García-Lobato, M. A.; de la Torre-Saenz, L.; Alonso-Lemus, I. L.; Rodríguez-Varela, F. J. Energy Generation from Pharmaceutical Residual Water in Microbial Fuel Cells Using Ordered

Mesoporous Carbon and Bacillus subtilis as Bioanode. ACS Sustain. Chem. Eng. **2019**, 7, 14, 12179–12187. DOI: 10.1021/acssuschemeng.9b01281

(25) Carrillo-Rodriguez, J. C.; Garcia-Mayagoitia, S.; Perez-Hernandez, R.; Ochoa-Lara, M. T.; Espinosa-Magana, F.; Fernandez-Luqueno, F.; Bartolo-Perez, P.; Alonso-Lemus, I. L.; Rodriguez-Varela, F. J. Evaluation of the Novel Pd-CeO_2-NR Electrocatalyst Supported on N-doped Graphene for the Oxygen Reduction Reaction and its Use in a Microbial Fuel Cell. J. Power Sources **2019**, 414, 103-114. DOI: 10.1016/j.jpowsour.2018.12.087

(26) Yang, J.; Tian, C.; Wang, L.; Fu, H. An Effective Strategy for Small-Sized and Highly-Dispersed Palladium Nanoparticles Supported on Graphene with Excellent Performance for Formic Acid Oxidation. J. Mater. Chem. **2011**, 21, 3384-3390. DOI: 10.1039/C0JM03361H

(27) Jiang, Y.; Lu, Y.; Li, F.; Wu, T.; Niu, L.; Chen, W. Facile Electrochemical Codeposition of "Clean" Graphene–Pd Nanocomposite as an Anode Catalyst for Formic Acid Electrooxidation. Electrochem. Commun. 2012, 19, 21-24. DOI: 10.1016/j.elecom.2012.02.035

(28) Li, Y.; Yu, Y.; Wang, J.-G.; Song, J.; Li, Q.; Dong, M.; Liu, C.-J. CO Oxidation over Graphene Supported Palladium Catalyst. Appl. Catal. B: Environ. **2012**, 125, 189-196. DOI: 10.1016/j.apcatb.2012.05.023

(29) Siamaki, A. R.; Khder, A. E. R. S.; Abdelsayed, V.; El-Shall, M. S.; Gupton, B.F. J. Catal. **2011**, 279, 1-11. DOI : 10.1016/j.jcat.2010.12.003

(30) Ferreira, J. C., Cavallari, R.V., Bergamaschi, V.S., M. Antoniassi R., Teixeira-Neto A.A., Linardi M., M. Silva J. C. Palladium nanoparticles supported on mesoporus biocarbon from coconut shell for ethanol electro-oxidation in alkaline media. Mater. Renew. Sustain. Energy. **2018,** 7, 23. DOI: 10.1007/s40243-018-0130-z

(31) Zhu, C.; Yang, Y.-Y.; Zhao, Z.-G. Surface Voltammetric Dealloying Investigation on PdCu/C Electrocatalysts Toward Ethanol Oxidation in Alkaline Media. J. Nanopart. Res. **2018**, 20, 314. DOI: 10.1007/s11051-018-4423-z

(32) Shafaei Douk, A.; Saravani, H.; Noroozifar, M. Novel Fabrication of PdCu Nanostructures Decorated on Graphene as Excellent Electrocatalyst Toward Ethanol Oxidation. Int. J. Hydrogen Energy **2017**, 42, 15149-15159. DOI: 10.1016/j.ijhydene.2017.04.280

(33) Rajesh, D.; Indra Neel, P.; Pandurangan, A.; Mahendiran, C. Pd-NiO Decorated Multiwalled Carbon Nanotubes Supported on Reduced Graphene Oxide as an Efficient Electrocatalyst for Ethanol Oxidation in Alkaline Medium. Appl. Surf. Sci. **2018**, 442, 787-796. DOI: 10.1016/j.apsusc.2018.02.174

(34) Ipadeola, A. K.; Lisa Mathebula, N. Z.; Pagliaro, M. V.; Miller, H. A.; Vizza, F.; Davies, V.; Jia, Q.; Marken, F.; Ozoemena, K. I. Unmasking the Latent Passivating Roles of Ni(OH)2 on the Performance of Pd–Ni Electrocatalysts for Alkaline Ethanol Fuel Cells. ACS Appl. Energy Mater. **2020**, 3, 9, 8786–8802. DOI: 10.1021/acsaem.0c01314

(35) Shen, S.; Guo, Y.; Luo, L., Li, F.; Li, L.; Wei, G.; Yin, J.; Ke, C.; Zhang, J. Comprehensive Analysis on the Highly Active and Stable PdAu/C Electrocatalyst for Ethanol Oxidation Reaction in Alkaline Media. J. Phys. Chem. C **2018**, 122, 3, 1604–1611. DOI: 10.1021/acs.jpcc.7b10009

(36) Hu, C.; Cheng, H.; Zhao, Y.; Hu, Y.; Liu, Y.; Dai, L.; Qu, L. Newly-Designed Complex Ternary Pt/PdCu Nanoboxes Anchored on Three-Dimensional Graphene Framework for Highly Efficient Ethanol Oxidation. Adv. Mater. **2012**, 24, 5493. DOI: 10.1002/adma.201200498
